# Challenges and opportunities in exosome research—Perspectives from biology, engineering, and cancer therapy

**DOI:** 10.1063/1.5087122

**Published:** 2019-03-27

**Authors:** Xia Li, Alexander L. Corbett, Erfan Taatizadeh, Nishat Tasnim, Jonathan P. Little, Cathie Garnis, Mads Daugaard, Emma Guns, Mina Hoorfar, Isaac T. S. Li

**Affiliations:** 1 Department of Chemistry, University of British Columbia, Kelowna, British Columbia V1V 1V7, Canada; 2 School of Engineering, University of British Columbia, Kelowna, British Columbia V1V 1V7, Canada; 3 School of Health and Exercise Sciences, University of British Columbia, Kelowna, British Columbia V1V 1V7, Canada; 4 Department of Integrative Oncology, BC Cancer Agency, Vancouver, British Columbia, V5Z 1L3, Canada, and Department of Surgery, University of British Columbia, Vancouver, British Columbia V5Z 1M9, Canada; 5 Vancouver Prostate Centre, Vancouver, BC V6H 3Z6, Canada, and Department of Urologic Sciences, University of British Columbia, Vancouver, Vancouver, BC V5Z 1M9, Canada

## Abstract

Exosomes are small (∼30–140 nm) lipid bilayer-enclosed particles of endosomal origin. They are a subset of extracellular vesicles (EVs) that are secreted by most cell types. There has been growing interest in exosome research in the last decade due to their emerging role as intercellular messengers and their potential in disease diagnosis. Indeed, exosomes contain proteins, lipids, and RNAs that are specific to their cell origin and could deliver cargo to both nearby and distant cells. As a result, investigation of exosome cargo contents could offer opportunities for disease detection and treatment. Moreover, exosomes have been explored as natural drug delivery vehicles since they can travel safely in extracellular fluids and deliver cargo to destined cells with high specificity and efficiency. Despite significant efforts made in this relatively new field of research, progress has been held back by challenges such as inefficient separation methods, difficulties in characterization, and lack of specific biomarkers. In this review, we summarize the current knowledge in exosome biogenesis, their roles in disease progression, and therapeutic applications and opportunities in bioengineering. Furthermore, we highlight the established and emerging technological developments in exosome isolation and characterization. We aim to consider critical challenges in exosome research and provide directions for future studies.

## I. INTRODUCTION

Interest in exosome research has increased dramatically in recent years due to their unique functions as intercellular messengers, abilities to alter recipient cell bioactivities, as well as therapeutic potential in disease diagnostics and targeted drug delivery.^1–3^ Exosomes are a type of extracellular vesicles (EVs) with diameters of 30–140 nm and are secreted from most cell types into the extracellular space after fusion of multivesicular bodies (MVBs) with the cell membrane.^4^ Alongside exosomes, cells secrete other types of EVs including apoptotic bodies (50–500 nm; released during apoptosis) and ectosomes (30–100 nm; assembled and released directly from the plasma membrane).^1,5–7^ Some of these EVs are similar to exosomes in their physical properties such as size and density, which makes isolating exosomes quite challenging.^8^ The primary difference among the various EVs is thought to be their particular mode of biogenesis, which in turn determines the cargo contents and functions.^5^ Non-exosomal EVs result from direct budding of plasma membranes whereas exosomes originate from the inward budding of endosomes into MVBs. From there, some MVBs are directed into the lysosomal compartment for degradation and recycling, while others form the intraluminal vesicles (ILVs) to be secreted outside of cells into body fluids as exosomes. During this process, parent cell information in the form of lipids, proteins, and nucleic acids is packed into exosomes which then can manipulate the functions of recipient cells on arrival.^9^ The content of the exosomes is therefore specific to the cell of origin, allowing parent cell signals to be transmitted to neighboring cells without direct cell to cell contact. Irrespective of the parent cell, exosomes share common features such as certain tetraspanins (CD9, CD63, and CD81), heat shock proteins (Hsp 60, Hsp 70, and Hsp 90), biogenesis related proteins (Alix and TSG 101), membrane transport and fusion proteins (GTPases, annexins, and Rab proteins), nuclear acids (mRNA, miRNA, and long non-coding RNAs and DNAs) , and lipids (cholesterol and ceramide).^1,10^ These unique properties of exosomes provide opportunities for innovations in diagnosis and treatments. For example, exosomes may contribute to the propagation of certain diseases including cancer metastasis. Investigation of the exosome content, biogenesis, and release mechanisms will not only improve our understanding of certain diseases but will also allow researchers to better target them for treatment. Moreover, researchers could utilize exosomes as natural drug delivery vehicles for increased targeting accuracy and decreased minimum dosage and side effects.

Despite significant effort into this relatively new field of research, our understanding of exosomes remains limited by factors including inefficient separation methods, lack of exclusive biomarkers, and lack of high-resolution visualization techniques. This review aims to summarize the current knowledge on exosome biogenesis and biological functions, as well as existing applications in therapy and emerging techniques in exosome characterization and isolation. Moreover, the limitations that hinder exosome research in isolation, purification, and characterization will be identified. Lastly, we hope to point out directions for future studies.

## II. BIOGENIC PATHWAY

Exosomes, by definition, differ from other types of EVs in their biogenesis. Whereas microvesicles are formed from the budding of the cell membrane, exosomes are the result of endosomal plasma membrane invagination during the process of endosomal maturation from early to late endosomes.^11^ These late endosomes, also known as multivesicular bodies (MVBs), contain a population of intraluminal vesicles (ILVs) that are called exosomes when released. MVBs are either transported to the cell membrane, with which they fuse and release their contents to the extracellular environment, or are transported to a lysosome and are digested. Hypoxic and genotoxic stresses, as well as the expression of activated oncogenes, on the cell induce exosome secretion through regulation of p52, though it is unknown whether this also increases ILV formation.^12^ Additionally, upregulation of the six-transmembrane epithelial antigen of prostate 3 (STEAP3), syndecan-4, and NadB has been used to increase exosome production by 15–40 fold in cell cultures.^13^ The exact mechanisms for the entirety of these processes have not yet been completely elucidated and much of the current knowledge arises from knockdown procedures, which do not provide full mechanistic insight Moreover, the processes of exosomal biogenesis use highly conserved complexes which have been given different names depending on their origin (i.e., yeast or metazoan origin); in this section, we will use the metazoan names whenever possible.

### A. ESCRT-mediated pathway

**FIG. 1.**
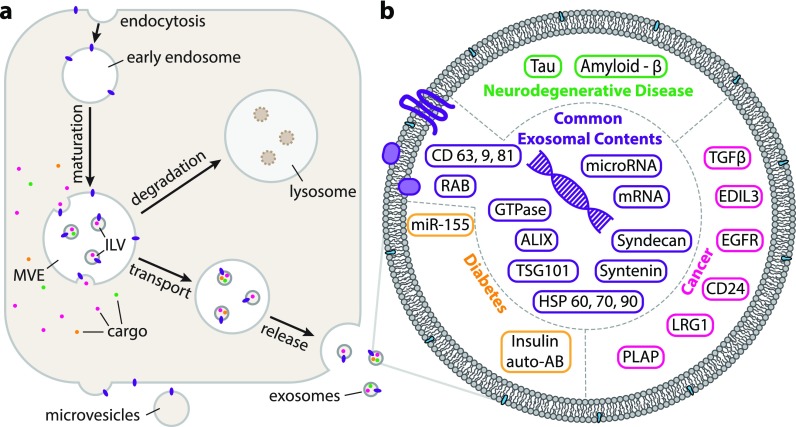
(a) Schematic representation of the major components of the endosomal pathway and the generation of exosomes. Components of the biogenic pathway may be redirected for degradation in the lysosome. (b) The contents of exosomes may serve as biomarkers for disease—some of the known ones, along with the standard exosomal biomarkers are categorised here. In addition to proteins, exosomes also contain many species of nucleic acids. The ESCRT machinery is not internalised.

The first step in the exosomal biogenic pathway is the formation of ILVs from the limiting membrane of maturing endosomes. The most notable complexes in the formation of ILVs are the Endosomal Sorting Complexes Required for Transport (ESCRT), a family of roughly two dozen proteins forming five complexes (ESCRT 0-III, Vps4) [Fig. 1(a)].^14^ Discovered in the early 2000s, the ESCRT complexes were determined to have a range of functions including cargo sorting and membrane remodeling in a collection of cellular processes of which ILV formation and cellular abscission during cytokinesis are the most studied.^15^ This process starts with ESCRT-0, which interacts with phosphatidylinositol 3-phosphate (PI3P) rich membrane regions and binds ubiquitinated cargo via Zinc Finger Domains (ZFDs) and Ubiquitin-interacting Motifs (UIMs), respectively.^16^ The ESCRT-0 complex has been described as both a heterodimer and heterotetramer of Hrs and STAM, which are constitutively bound to one another, and is able to weakly associate with up to eight different ubiquitin moieties simultaneously, using the Double Ubiquitin Interacting Motif of Hrs as well as the (Vps27/Hrs/STAM) domain and UIM of STAM, in the case of the heterotetramer.^17^ There remains no known method for the selection of ubiquitinated cargo but an answer might lie in their attachment to clathrin coats prior to sequestration.^18^ Next, a domain in the C-terminus of the Hrs subunit of ESCRT-0 recruits ESCRT-I.^16^ This complex is a heterotetramer of the Tumour Supressing Gene 101 (TSG101), Vacuolar protein sorting associated proteins Vps28, Vps37, and multivesicular body (MVB)12 all curled to form a rod-like structure with domains for ESCRT-0 and ESCRT-II at the opposing ends.^15^ In fact, ESCRT-I and ESCRT-II appear to exist as a supercomplex^19^ that induces the budding of the endosome away from the cytoplasm.^20^ During this budding process, the ESCRT-0 bound cargoes are relocated to the bud along with any other cargoes the loading system selects. Following bud formation and cargo selection, the Charged Multivesicular Body Protein (CHMP) 6 of the ESCRT-III complex binds directly to the ESCRT-II complex which activates the recruitment of CHMP4. This protein polymerizes as a coil around the neck of the budding ILV and serves as the drawstring to the ILV pouch, which is capable of drawing closed with the addition of CHMP3 completing ESCRT-III assembly.^20^ This is made possible by the high affinity the ESCRT-III subunits have for the plasma membrane. Following that a Vps4 complex (formed by SKD1, LIP5, and CHMP5) is required for the disassembly of ESCRT-III, a process that is completed by pulling on the ESCRT-III polymer and unfolding each individual protein sequentially.^21,22^ This is the only process in ILV formation to have been characterized as adenosine triphosphate (ATP)-dependant. It has been noted that the ESCRT-III complex readily recruits deubiquitinating enzymes that sever the weak connection between the ESCRT-0 complex and the cargoes localized at the ILV lumen; a process which recycles ESCRT-0 for use elsewhere in the cell.^16^ There is no consistent internalization of the ESCRT machinery into ILVs.

### B. ESCRT-independent pathway

In addition to ESCRT mediated ILV formation, an ESCRT-independent pathway exists, though these pathways may not be mutually exclusive and have been proposed to work in tandem. The mechanisms of the ESCRT-independent pathway are not entirely clear and may be numerous with sorting and budding mechanisms being found that are independent of well-established ceramide-mediated membrane budding.^23^ When ceramide is, however, used it is produced by the breakdown of sphingomyelin by neutral sphingomyelinase and forms raft-like structures due to its ability to self-associate.^24^ This associated, coupled with the conical shape of the lipid, is assumed to drive the initial deformation of the membrane.^18^

### C. Loading mechanisms and cargo

The main interest in exosomes remains their potential use as biomarkers of disease and as vessels for drug delivery. To this end, knowledge regarding their contents and the loading mechanism is of great value. However, these mechanisms are still poorly understood.

Considering the trafficking of membrane proteins, the ALG-2 Interacting Protein X (ALIX) has been noted to bind to the ESCRT machinery and to Syntenin-1, which subsequently binds to syndecans or CD63 via a PDZ domain.^25^ Current models for the sequestering of cargo include the association of specific cargoes to the heparan sulfate (HS) proteoglycans (HSPGs) of syndecan, which clusters following the trimming of the heparan sulfate by heparanase.^26^ A conveyor belt model in which ubiquitinated cargoes are passed along the ESCRT chain to the budding membrane with sequential association with UIMs from different downstream components of the ESCRT complexes has also been proposed.^16^ Both of these may prove to be valid mechanisms. Regardless of the mechanism, multiple proteins are consistently identified as exosome constituents and of these, ALIX, TSG101, and CD63 are commonly employed as markers^27^ along with the tetraspanins CD60, CD9, and CD81 [Fig. 1(b)].^28^ The fidelity of any marker, however, is dependent on the cell type of origin as exosomes are a heterogeneous population expressed differently by different cells; where professional antigen presenting cells will release exosomes displaying Major Histocompatibility Complex (MHC) class II proteins, tumour cells will release exosomes presenting tumour antigens.^29^ Cells even express a heterogeneous exosome population with both distinct protein and RNA compositions.^27^ Willms *et al.* demonstrated this by isolating exosomes with ultracentrifugation (UC) followed by loading them from the top and bottom on a discontinuous sucrose gradient and centrifuging. While this indicated two different exosome populations, there is no indication as to why these populations are different or where they differed in their biogenesis.

In addition to proteomic cargoes, exosomes carry genetic materials including miRNA, various non-coding RNAs, mitochondrial RNAs, and mRNAs [Fig. 1(b)].^30^ The mechanisms for loading these cargoes is not yet known, though it has been proposed that RNA cargo associates with sphingomyelin and cholesterol enriched regions of the budding membrane prior to bud formation.^31^ A different model involves the sorting of RNA by sumoylated hnRNPA2B1 via the presence of a “zip code” in the 3′UTR of mRNA.^32,33^ Conversely, it has been noted that exosomal RNA cargo reflects the state and cytoplasmic content of the cell of origin.^34^ Regardless of the loading mechanism, it has been determined that exosomes provide a method to exchange genetic information between cells.^35^ Considered the main functional component of the exosome, once in the recipient cell, RNA plays the role it would in the cell of origin (e.g., miRNA repressing target mRNA).^36^ That said, the RNA transported by exosomes is not always native to the cell; infected cells have been noted to produce exosomes containing RNA of viral origin which, upon uptake, infects the recipient cell.^37^ An extreme example of this can be seen with the Human Immunodeficiency Virus (HIV) for which it has been postulated that the membrane casing of the virus is in fact a hijacked exosome carrying viral RNA.^38^

Unlike RNA and proteomic sorting mechanisms, lipid sorting is not a large area of study and thus relatively devoid of information. The process is possibly driven by specific pH differences and the resulting modifications of lysobisphosphatidic acid (LBPA), lysophosphatidylcholines, and phosphatidic acids.^39,40^ Particularly, phosphatidylserine has been noted to be mildly enriched in exosomes relative to MVBs.^40^

### D. Transport and release

Following ILV formation, the MVB is either transported to and digested in a lysosome or pulled along microtubule tracks for fusion with the plasma membrane. The mechanism that selects which MVBs to degrade and which to not, is unknown. However, it is known that increasing the ISGylation of TSG101 on the MVB decreases MVB populations and consequently the number of exosomes released.^41^ Additionally, cortactin, a protein responsible for stabilizing actin, has been positively related to secretion without modifying the cargo content.^42^ MVBs marked for degradation have also been shown to be deficient in cholesterol relative to those that fuse with the plasma membrane.^43^ In the trafficking of vesicles, the Rab GTPases act to recognise the acceptor membranes and bind to tether proteins.^44^ This is followed by the associated of glutamine (Q) soluble NSF attachment protein receptor (SNARE) and arginine (R)-SNARE proteins, also commonly known as vesicle (v)-SNARE and target (t)-SNARE, which bring the membranes into close proximity. Specific to the exosomal release pathway, Rab27 and Rab35 specialize in the transport and docking of the MVB to the plasma membrane^45,46^ while Rab11 is involved in membrane abscission.^47^ Knockdown experiments performed by Ostrowski *et al.* suggested that Rab27b controlled the transfer of MVBs from microtubule tracks to the actin of the cell cortex, while Rab27a mediated docking with the plasma membrane.^48^ Down-regulation of the components responsible for the mechanical movement of MVBs to the plasma membrane results in a decrease in exosome release.

### E. Exosomes as biomarkers

The main interest in the application of exosome research is the possibility of using exosomes as biomarkers for disease and as delivery systems for therapeutics. This is of great importance due to their ability to cross the blood brain barrier.^49^ Additionally, the fluid biopsy required to analyse biomarkers in the blood or urine is minimally invasive. These also allow for the use of miRNA as a biomarker; previously, this was not possible as miRNA is easily degraded but these cargoes appear to be protected which allows for detection.

Cancer has been the subject of much investigation in exosome biology. Use of exosomes and their contents and surface proteins may allow earlier detection of cancers, which could increase prognosis and survival. The Canadian Cancer Society ranks breast cancer as the third highest cancer diagnosis nationally with diagnoses in stage I having a near 100% five-year survival rate compared to a 22% survival rate for breast cancers diagnosed in stage IV.^50^ The presence of CD24, EDIL3, and fibronectin proteins on circulating exosomes has been proposed to be markers of early stage breast cancers.^51^ For exosomes from non-small cell lung cancer, epidermal growth factor receptor (EGFR), placental alkaline phosphatase (PLAP), and leucine-rich alpha-2-glycoprotein 1 (LRG1) proteins were among those found to be overexpressed.^52,76^ Proteins do not need to be enriched in exosomes to be useful markers. Work done by Chen *et al.* with colorectal cancer patients found that exosomes from these individuals had decreased counts of HSP90, VTN, and MAPK1 among others as compared to healthy controls.^53^

Because of the ability of exosomes to cross the blood brain barrier, other biomarker studies relate to neurobiology. It has been demonstrated that spread of phosphorylated tau proteins occurs through an exosomal pathway prior to neural death in early Alzheimer's patients.^37^ Additionally, the amyloid β-proteins found in the plaques characteristic of Alzheimer's disease have also been noted to be expressed in exosomes while proteins characteristic of exosomes were found to be accumulated in said plaques.^54^ This suggests that exosomes play a role in the pathogenesis of Alzheimer's disease and that these proteins may serve as indicators of Alzheimer's disease if detected on exosomes. Additionally, Ebrahimkhani *et al.* compared the serum exosomal miRNAs of MS patients and healthy controls. They found that they could not only differentiate between those with and without MS, but also whether an MS patient's disease was relapsing or progressive.^55^

Another disease where exosome research may have a large impact is diabetes mellitus. Recently, it has been shown that a significant difference exists between the miRNA content of exosomes isolated from the serum of type 1 diabetes patients relative to an assumed healthy control group.^56^ The same study also observed that these exosomes resulted in a lower insulin output of islets in the presence of sugars suggesting that the exosome contents may play a role in the pathogenesis of type 1 diabetes. This study appears to be done in response to the lack of unique or useful biomarker for the disease. Though insulin autoantibodies, either free or associated with exosomes, may be used to identify future diabetes patients, these markers appear much too late in the development of the disease to be used with the aim of identifying potential patients and preventing disease development.^57^ Preclinical studies also indicate that exosomes may be involved in the pathogenesis of type 2 diabetes.^58^ The study showed that adipose tissue macrophages from obese mice release exosomes that are enriched in miR-155 and that these exosomes impair insulin sensitivity via novel inter-organ crosstalk with both the liver and muscles. These data suggest that exosomal miR-155 could be a biomarker related to insulin resistance and risk for type 2 diabetes.

Use of exosomes as biomarkers is not without its challenges. Cells produce similar sets of proteins and miRNAs while exosomes also express similar protein and RNA profiles. There are, however, few unique cell-specific proteins.^59^ Additionally, because the exosome populations expressed from single cells are heterogeneous, the content concentrations are expected to exist in a range and not at a set standard. Furthermore, exosome circulation in the body originates from a variety of different cell types and as such, unless they contain exceedingly distinct cargoes, it would be challenging to determine their tissue of origin. To date, there is a general lack of compiled data to be able to diagnose diseases based on exosomes alone. And, considering practical clinical limitations, there are currently no technologies for the detection and analysis of exosomes that are convenient in terms of the time spend to analyse a sample, sample throughput, quality control, inter-lab variability, and accuracy of the results. Prior to implementation of any diagnostic practice, a complete database of the exosomal profiles seen in diseases should be compiled to prevent misdiagnosis due to similar cargo contents, and technologies must be developed for a clinical setting.

## III. EXOSOMES AND CANCER

**FIG. 2.**
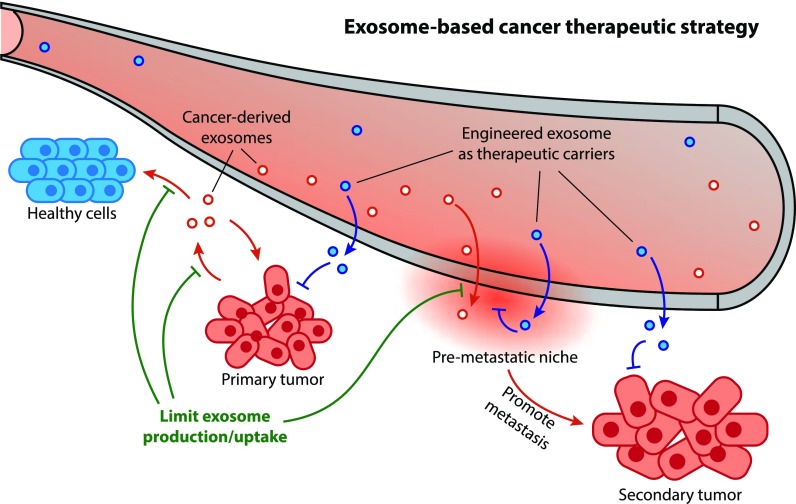
Schematic illustration of exosome's roles in cancer development and therapeutic application. Exosomes can facilitate tumor progression, establishment of pre-metastatic niche, and spreading to the secondary site (Sec. III). Exosome-based cancer therapy can be done by limiting exosome production/uptake (Sec. IV A) or utilizing exosomes as native gene/drug carriers (Sec. IV B).

Although exosome release is a normal process, cancer cells release exosomes at an elevated level and their cargos are particularly suitable for cancer progression.^10^ Once released from the cell, tumor exosomes start to circulate in extracellular space until they reach the targets. During this process, the cargo contents of exosomes are protected by the lipid bilayer membranes, shielding them from degradation by enzymes or other extracellular conditions. After being taken up by recipient cells, the tumor exosomes can alter the recipient cell function and phenotype, impacting both surrounding and distant non-tumor cells to promote a favorable microenvironment for cancer proliferation, dissemination, and metastasis (Fig. 2).^10,60–64^ Cancer progression is a complicated process and exosomes seem to be involved in every stage in the development. Subsections III A–III C highlight some of the key aspects where exosomes are involved in the evolution of cancer.

### A. Exosomes facilitating tumor proliferation and altering the microenvironment

Studies have reported that cancer derived exosomes promote tumor growth by directly activating the signaling pathways responsible for sustaining the tumor proliferation such as P13K/AKT (phosphorylated phosphatidylinositol 3-kinase/protein kinase B) or MAPK/ERK (mitogen-activated protein kinase/extracellular signal-regulated kinase).^65–68^ In gastric cancer cells, induction of cell proliferation was observed through activating P13K/AKT or MAPK/ERK pathways mediated by exosomes.^66^ Another study of gastric cancers confirmed the exosomal CD97 was responsible for mediating proliferation through the MAPK pathway.^67^ In addition, exosomes from bladder cancer and oral squamous carcinoma cells have been shown to induce cell proliferation via the PI3K/AKT and the MAPK/ERK pathways.^68,69^

In addition to the effects on cell proliferation, tumor cell derived exosomes can alter the microenvironment to facilitate invasion and dissemination of the disease. Particularly, exosomes derived from prostate cancer cells were shown to turn fibroblasts into activated fibroblasts or myofibroblasts by delivering Transformation Growth Factor beta (TGFβ) to the extracellular milieu.^70,71^ Fibroblasts are dominant components in tumor tissues, and their active form is well described for their role in tumor progression by the secretion of growth factors.^72,73^ Similarly, fibroblasts in bladder cancer were triggered to differentiation and activation by exosome-mediated TGFβ transfer.^74^ Another example of exosomes manipulating the tumor microenvironment can be found in the induction of the angiogenesis process.^75,76^ Angiogenesis is a natural occurring process forming blood vessels using pre-existing vessels, and it is common in organisms during growth and development, as well as in response to injury.^75,76^ However, this process is also essential in cancer progression since tumor growth requires rapid forming of vasculature to provide access to nutrients, oxygen and waste removal.^75^ Several studies have found that exosomes play an important role in angiogenesis through the transfer of miRNA, mRNA, and proteins.^77,78^ For example, Umezu *et al.* reported leukemia cells derived exosomes overexpress miR-92a (i.e., a miRNA that belongs to mir-17–92 cluster) entering endothelial cells and resulting in an enhanced migration and tube formation.^77^ Additionally, Delta-like 4 (Dll4), a membrane-bound Notch ligand with a fundamental role in vascular development and angiogenesis, can be transported via exosomes through the 3D collagen matrix and to distant cells.^78^

### B. Exosomes and metastasis

Besides altering the local tumor microenvironment to promote cancer proliferation, tumor derived exosomes are shown to facilitate metastasis at distant organs.^60–62^ Tumor metastasis is a multi-step process including detachment from the primary organs, invasion and migration through the basement membrane, dissemination through the blood stream, and finally adaption and colonization to the secondary organ sites.^79,80^

Cancer cells have developed exosome mediated strategies to influence numerous steps in metastasis. For instance, triple negative breast cancer cell lines (MDA-MB-231) overly express miR-10b, and the derived exosomes can transfer miR-10b to non-malignant Human Mammary Epithelial cell line, subsequently inducing the invasion ability.^81^ Exosomes derived from epithelial ovarian cancer (EOC) cells were shown to enhance ovarian cancer invasion by transferring CD44 to human peritoneal mesothelial cells (HPMCs).^82^ The HPMC cells are a single layer of cells lining the peritoneal cavity where EOC first attach to during metastasis. Upon receiving CD44 from EOC exosomes, HPMC cells are reprogramed to change to a prometastatic spindle phenotypes to support EOC invasion and metastasis.^82^ Additionally, miR-105 from metastatic MDA-MB-231 exosomes can target the tight junction protein (ZO-1), destroy the endothelial cell barriers, induce vascular permeability, and promote metastasis *in vivo*.^83^

Following invasion and intravasation, cancer cells could modulate the microenvironment of the distant organ to allow survival and colonization for tumor cells prior to their arrival.^84^ These predetermined microenvironments are called “pre-metastatic niche” and the formation of such a phenomenon can be introduced by exosomes from cancer cells.^62,84–86^ It was shown that pancreatic ductal adenocarcinoma (PDAC) derived exosomes induce pre-metastatic niche formation in the liver and naive mice treated with exosomes from PDAC had an increase in the liver metastatic burden.^62^ The study also investigated the mechanisms involved in the process and revealed the uptake of exosomes by liver Kupffer cells induced the production of TGFβ, which upregulates fibronectin production by hepatic stellate cells and influx of bone marrow-derived macrophages.^62^ The cargo contents of PDAC derived exosomes include a high expression of the macrophage-inhibitory factor (MIF). Consequently, blocking MIF in exosomes resulted in a decrease in TGFβ, fibronectin deposition, macrophage formation, and metastasis liver burden.^62^ Melanoma exosomes are shown to condition remote lymph nodes to facilitate formation of the pre-metastatic niche.^86^ In particular, melanoma exosomes, home to sentinel lymph nodes, influence the lymph node distribution pattern of free melanoma cells, and enhance cell migration to exosome rich sites. The upregulation of genes was involved in cell recruitment, extracellular matrix remodeling, and vascular proliferation factors, all of which contribute to the establishment of a microenvironment that favors melanoma cell recruitment and colonization.

### C. Exosomes and cancer immune systems

Tumor-derived exosomes are also known to have significant impacts on the immune system in cancer development.^87–91^ On one hand, tumor-derived exosomes can stimulate immune response against cancer, also known as cancer immunosurveillance.^89,92,93^ For example, tumor derived exosomes contain and deliver tumor antigens to dendric cells produce exosomes, which in turn stimulate T-cell-mediated anti-tumor immune response.^91^ Therefore, specific exosomes-containing cancer antigens are being studied as cancer vaccines in immunotherapy.^87,93^ On the other hand, tumor-derived exosomes can facilitate immunosuppression and inhibit immunosurveillance in order to invade and spread.^90,92^ Indeed, the cargos of many tumor derived exosomes contain molecules from the parent tumor cells that can directly or indirectly influence the activation, development, and antitumor activities of immune cells.^88,90,92^

## IV. EXOSOME-BASED THERAPEUTIC OPPORTUNITIES

Owing to their essential roles in disease propagation, cancer proliferation and metastasis, exosomes have been investigated as promising therapeutic platforms. Indeed, targeting disease derived exosomes allows us to control the spread and progression of certain illnesses.^18,94,95^ Alternatively, exosomes' native structure and the unique cellular functions provide great potential as natural carriers for therapeutic molecues.^96–99^ Sections IV A and IV B detail the applications in utilizing exosomes as therapeutic targets and natural drug/gene delivery vehicles.

### A. Inhibit disease derived exosomes

To diminish the number of disease derived exosomes expressed, many studies have turned their attention on exosome biogenesis pathways and explored how to block certain pathways to reduce exosome production, release and uptake. For example, ceramide was identified as one of lipids in the ESCRT-independent biogenesis pathways and its synthesis is mediated by neutral sphingomyelinase 2 (nSMase2).^1,18,100^ As a result, many treatments tried to reduce ceramide production by either knocking down nSMase2 genes or adding the nSMase2 neutral inhibitor GW4869 to ultimately eliminate or reduce exosome production.^81,94,101^ A study on treating inflammatory disease, sepsis, injected GW4869 to wild-type mice and observed significant impaired release of both exosomes and pro-inflammatory cytokines, which were possibly mediated by exosomes.^94^ The therapeutic role of nSMase2 was shown in treating Alzheimer's diseases, where exosomes are involved in spreading the tau protein, a hallmark of AD.^101^ The nSMase2 was either silenced with short interfering RNA or inhibited using GW4869, and the results showed the tau protein secretion was significantly reduced with both methods.^101^ GW4869 treatment also decreased miR-10b transfer in breast cancer, impairing miR-10b mediated cell proliferation in recipient cells.^81^

Targeting Rab proteins is a popular choice to limit exosome release due to their essentiality in exosome biogenesis and secretion.^1,45,48,102^ The small Rab GTPase belongs to the super family of Ras GTPases, and they are key regulators for intracellular transport.^45,48,103^ In particular, Rab 27a and 27b are involved in MVB docking and exocytosis in HeLa cells, and therefore inhibition of Rab 27a or Rab 27b has been shown to reduce exosome release.^48^ In lung cancer cells (A549) and carcinoma cells (4T1 and TS/A), exosome secretions were impaired when Rab 27a was suppressed by shRNA.^104,105^ Furthermore, Rab 27a inhibition resulted in reducing growth in primary tumors and decreased metastasis in 4T1 carcinoma cells.^105^ Similarly, Rab 11 was also shown to be involved in docking and fusion of MVBs, as well as exosome release in a calcium dependent manner in leukemia cells.^102^ Rab11-overexpression resulted in MVBs accumulation in the plasma membrane in the presence of a calcium chelator. As a result, the inhibition of Rab 11 lead to a reduction of calcium mediated exosome decrease.^102^

Blocking exosome uptake pathways have also been exploited to stop the dissemination and spread of diseases.^106,107^ Exosome internalization mechanisms are extremely diverse including clathrin-mediated endocytosis, phagocytosis, micropinocytosis, and plasma or endosomal membrane fusion, thus providing opportunities to target various components in exosome uptake pathways.^4,106^ Heparan sulfate (HS) proteoglycans (HSPGs) are family of proteins that have shown to function as internalizing receptors for exosomes to adhere and to internalize.^107^ Heparin, as an HS mimetic, inhibit exosome uptake in a dose, size, and overall sulfation charge dependent manner and significantly reduced glioblastoma cell migration.^107^ Dynamin proteins have been described as essential mediators in the Clathrin-Mediated Endocytosis pathway, and it can be effectively inhibited by dynasore.^108,109^ Uptake of Mantle cell lymphoma exosomes was significantly inhibited by Dynasore, suggesting a potential effective treatment for aggressive and incurable lymphoma.^110^ In melanoma cells, dynasore treatment suppressed exosome internalization in normal endothelial cells, as well as blocked tumor exosome induced phenotypic changes in favor of the tumor microenvironment in endothelial cells including activation of the P13K/Akt pathway, enhanced cell migration, and angiogenesis.^109^

### B. Using exosomes as therapeutic platforms

An ideal delivery vehicle for therapeutic treatments should be specific to the targeting sites with low toxicity to other organs, high encapsulation, and delivery efficiencies, protects the payload while in circulation, and maintains a steady release profile.^111–113^ In recent decades, polymer-based nanocarriers with specific targeting molecules have been developed, and the materials include block or alternating copolymers, cationic polymers, and liposomes.^114–119^ However, these systems often suffer from challenges such as drugs preferential accumulation in the spleen and liver tissues instead of disease sites,^120,121^ cytotoxicity of the polymer materials,^122,123^ and multi-drug resistance developed by cancer cells over time.^123^ To that end, exosomes may be ideal cell transporters to deliver drugs/nucleic acids, providing several advantages over the polymer-based delivery methods. First of all, exosomes are naturally present in body fluids so that they are stable under both physiological and pathological conditions. For example, immune related miRNA are found in exosomes derived from human breast milk, and they are shown to be very stable in very acidic conditions, thus tolerating an infant's gastrointestinal environment.^124^ Second, exosomes are less toxic and immunogenic compared to other nanocarriers especially when obtained from immature dendritic cells and monocytes.^87,125,126^ Third, exosomes could reduce the multi-drug resistance that other nanocarriers face by transferring the proteins or miRNAs that modulate thee resistance phenotype to recipient cells.^87^ Additionally, exosomes are driven to deliver cargo to specific recipient cells, due to the unique membrane proteins and lipids that can bind to specific receptors at the recipient cells, thus enhancing the delivery efficiency.^96^ Finally, exosomes are able to cross the blood-brain barrier (BBB), a major challenge in drug delivery research as most drugs and carriers cannot cross this barrier, which makes exosomes an excellent choice to deliver cargos to the brain.^49,127^

#### 1. Exosomes as gene carriers

Naked therapeutic genes cannot cross the plasma membranes effectively due to their high molecular weight and negative charge, making the cellular uptake very limited.^117,128,129^ Undecorated novel genes are also at risk of being rapidly degraded by nucleases, making them inaccessible to the targeting cell. Therefore, the success of gene therapy is largely dependent on the development of the gene delivery vectors. Both viral and non-viral carriers have been developed as gene delivery vehicles, however, they have major drawbacks such as a high systematic toxicity and trigger immune response.^129^ Alternatively, exosomes are superior gene carriers since they are biocompatible, immunologically inert when sourced properly, and could reach the target efficiently.^130^

To load nucleic acids into exosomes, the therapeutic materials can be transfected into donor cells, thus packaged into exosomes subsequently or can be incorporated into exosomes post-isolation through electroporation.^96,97,131^ For example, the therapeutic mRNA/protein, CD-UPRT-EGFP (CD-cytosine deaminase; UPRT-uracil phosphoribosyl transferase; and EGFP-enhanced green fluorescent protein) were pre-transfected into donor cells prior to harvesting exosomes.^131^ The recipient schwannoma cells were treated with these exosomes along with pro-drug 5-FC (5-fluorocytosine), which was converted an active anti-cancer drug 5-FU (5-fluorouracil) by CD and UPRT.^131^ The engineered exosomes targeted the tumor cells effectively and showed regression of tumor growth.^131^ Wood laboratory proposed another effective loading mechanism by transfecting donor cells with plasmids expressing specific targeting proteins and incorporating desired nucleic acids into exosomes through electroporation.^132^ The first study that successfully adopted this method was by Alvarez-Erviti *et al.*, where immunologically inert exosomes were produced by self-derived immature dendric cells.^96^ The surface of exosomes was engineered to express an exosomal membrane protein Lamp2b, fused with a brain specific rabies virus glycoprotein (RVG) peptide to increase the targeting capacity. After purification, RVG exosomes were loaded with siRNA and delivered *in vitro* and *in vivo.*^96^ The results showed that exosomes can cross the BBB and deliver therapeutic siRNA safely and effectively into the brain with little toxic effects or immunogenicity even after repeated dosage.^96^

#### 2. Exosomes as drug delivery carriers

Drugs are generally encapsulated into purified exosomes by methods such as incubation, sonication and electroporation.^133,134^ These loading mechanisms were tested in a study, where chemotherapeutic drugs paclitaxel (PTX) were encapsulated in macrophage derived exosomes (exoPTX), and the results from this particular study revealed sonication was shown to be the most effective method.^123^ In addition, exoPTX showed preferential accumulation in cancer cells and decreased metastasis compared to liposomes and polymer-based carrier counterparts.^123^ Tian and colleagues engineered exosomes with targeting proteins to deliver doxorubicin (Dox) specifically to breast cancer cell lines.^98^ Similar to the pioneer work done by Alvarez-Erviti *et al.*,^96^ exosomes were generated in immature dendritic cells to reduce immunogenicity and toxicity, and the iRGD-Lamp2b plasmid was transfected onto the cell lines to express Lamp2b (exosomal membrane protein) fused to the αv integrin-specific iRGD peptide for enhanced targeting effects.^98^ After purification, Dox was encapsulated into exosomes through electroporation and applied to breast cancer cell lines. The results showed that engineered exosomes were able to deliver Dox specifically to tumour tissues, leading to inhibition of the tumour growth with reduced toxicity and immunogenicity.^98^ Exosomes are also used to encapsulate curcumin, a natural hydrophobic polyphenol therapeutics from turmeric that has anti-inflammatory, anti-neoplastic, and anti-cancer properties.^133^ Curcumin was incorporated into murine tumour cell line (EL-4) derived exosomes through incubation (exo-cur), followed by sucrose-gradient centrifugation. The exo-cur greatly improved curcumin stability and bioavailability, and *in vivo* results showed that mice treated with curcumin complexed with exosomes are protected against lipopolysaccharide (LPS)-induced septic shock. In a follow up study, exo-cur were selectively taken up by microglial cells in the brain via intranasal administration, demonstrating a non-invasive and effective treatment of inflammatory brain diseases bypassing the BBB.^135^ In this study, numerous cell types including normal mouse embryo fibroblast cells and tumor cells were used to produce exosomes, and the exo-cur were found to reduce the numbers of inflamed microglial cells after 2 h, along with an increase in apoptotic events.^135^

Considerable efforts have been made to exploit exosomes as targeted therapeutic carriers (examples of which are found in Table I). However, there are major challenges that the future studies need to address. First, a major limitation in this field is the lack of standardized techniques for the isolation and purification of exosomes. The conventional methods of isolation require multi-step ultracentrifugation. However, this method is tedious, and the obtained exosomes are often contaminated with other types of EVs. As the targeting and delivery abilities of exosomes are essential in drug/gene delivery applications, the presence of non-exosomal EVs will hinder the therapeutic efficiencies. Developing a fast and precise method of exosomes isolation is therefore one of the most important tasks in the current field of research. Second, the cell origins of exosomes need to receive more attention for specific applications. For example, in utilizing exosomes for cancer therapeutics, one should avoid sourcing exosomes from cancer cells, as they may contain oncogenic drives that may contribute to cancer progression. Additionally, exosomes have been derived from many different human cell types, though the way that cell types may affect exosomes delivery properties remains unknown.^127^ Therefore, thorough and precise characterization studies of exosomes are needed before applying exosomes as therapeutic carriers. Finally, exosomes extracted from cell cultures can vary and display inconsistent properties even when the same type of donor cells were used.^135^ Current cell culture and exosome purification technologies restrict the implementation of standardized and mass production of exosomes.^3^ Therefore, for exosomes to be considered as a reliable therapeutic platform, scalable manufacturing processes are needed to produce exosomes in a fast, reproducible, and cost-effective fashion.

**TABLE I.** Examples of exosomes used as drug/gene delivery carriers.

Donor cell origin	Therapeutic agents	Loading mechanisms	Targeting peptide	Targeting sites
Immature DC cells	siRNA	Electroporation	Lamp2-RVG	Mouse brain^96^
HEK293T	Suicide mRNA CD-UPRT-EGFP	Pre-transfected parent cells	NA	Schwannoma tumours^131^
Immature DC	Dox	Electroporation	Lamp2b-iRVG	Breast cancer^98^
HEK293	Let-7a miRNA	Transfection	GE11 or EGF	Breast cancer^97^
EL-4, MDA, 4T-1	Curcumin	Sucrose gradient centrifugation	NA	Multidrug Resistance (MDR) cell lines^133^
RAW 264.7	Paclitaxel	Incubation, electroporation, and sonication	NA	MDR cell lines^123^
PFSK-1 cells, bEND.3, A-123 and U-87 MG	Rhodamine 123, paclitaxel and doxorubicin	Incubation	NA	U-87 MG cells and zebra fish embryo^127^

In conclusion, exosomes have demonstrated great potentials in therapeutic applications. However, to advance in this field, a few fundamental obstacles need to be overcome. One of the most urgent hindrances is to develop reliable and efficient protocols for isolation and characterization. Sections V and VI review and discuss the current state of exosome isolation and characterization techniques.

## V. ISOLATION

Despite the fast growth in exosome research, isolation and purification techniques are still poorly developed and standardized.^59,136,137^ Exosome isolation from raw biological fluids is challenging as some components of biological fluids such as lipoprotein, chylomicrons, and microvesicles have size overlaps with exosomes (30–150 nm).^138,139^ Isolation from conditioned cell culture media is less complicated; however, other types of EVs are often co-isolated due to their size overlap and lack of specific biomarkers.^6,59^

Various techniques have been introduced for exosome purification and these methods all impact the yield, diversity, and functions of EVs recovered.^140–143^ Such techniques can be classified into two subgroups: conventional methods and microfluidics-based methods. The conventional methods such as ultracentrifugation, size exclusion chromatography, ultrafiltration, immunoaffinity, and polymer-based precipitation are established and widely used; however, they have not shown high efficiency or recovery yield.^144^ On the other hand, microfluidic devices are regarded as the emerging isolation platforms with numerous advantages such as: low sample consumption, high sensitivity, ease of use, and fast speed compared to conventional methods.^145–148^ Table II summarizes various conventional and microfluidics-based (referred to as emerging) exosome isolation techniques with their working principles, advantages, and disadvantages. In Secs. V A and V B, the details of these methods and their advantages and demerits are discussed.

**TABLE II.** Comparison of conventional and microfluidics-based exosome isolation techniques.

Protocols	Method of isolation	Isolation techniques	Working principle	Advantages	Disadvantages
Established	By density	Ultracentrifugation (UC) and gradient	Exerting sequential centrifugal forces on bioparticles based on the density, size, and shape differences	Easy to use, and long lifespan	Time-consuming process, low recovery and purity, and morphology changes
	By size	Size exclusion chromatography (SEC)	Large hydrodynamic radius likes exosomes cannot pass through these pores and excluded	Isolation without the presence of albumin in purified exosomes	Low recovery and purity
		Ultrafiltration (UF)	Trapping bioparticles based on the size differences by the nano-membranes	Higher purity as well as lower time consumption	Clogging problem in the nano-membrane, Exerting high shear stress on the bioparticles
	By function	Immunoaffinity	Fishing out exosomes based on the interaction of surface biomarkers (antigens) and immobilized antibodies	Higher recovery rate and purity compared to other conventional methods, suitable for specific type of exosomes	Large quantities of biological samples cannot be processed, high reagent cost, only cell-free samples can be used, low yield
		Polymer-based precipitation	Altering solubility or dispersibility of bioparticles by volume-excluding polymers	Large amount of sample can be processed, easy to use	Pre-and post-cleanup are required, lower efficiency of isolation due to co-precipitation of other non-exosomal contaminants
Emerging	Microfluidics-based		Isolation with miniaturized devices in various approaches such as acoustic, dielectrophoresis, filtration	High purity, low volume consumption, high sensitivity, reduced procedural costs, and sample-to-answer manner	Low isolation capacity, lack of global protocols and standardization, and high technical expertise is required

### A. Established protocols

Conventional methods of exosome purification have widely been used in the last decades in laboratories and clinics. These methods isolate exosomes either based on their physical properties (such as density and/or size) or their functions. Consequently, conventional methods can be classified into three subgroups: (1) density-based isolation, (2) size-based isolation, and (3) function-based isolation. Sections V A 1–V A 3 describe these methods, their working principles, and their advantages and disadvantages.

#### 1. By density

**FIG. 3.**
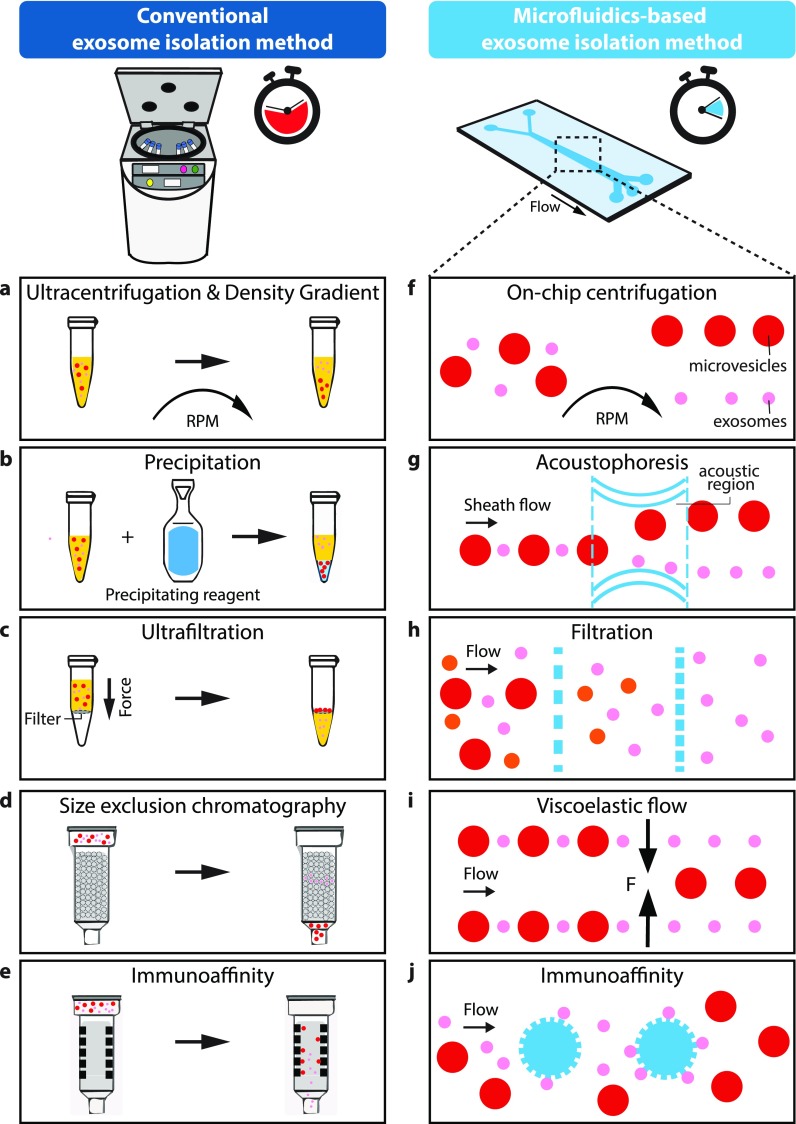
Schematic view and comparison of conventional (a)–(e) and microfluidic based (f)–(j) isolation methods commonly used to extract exosomes from biological fluids.

Isolation of exosomes based on their density can be accomplished by ultracentrifugation with and without a density gradient [Fig. 3(a)].^136,149^ In these methods, density differences between the medium and bioparticles as well as among bioparticles provide opportunities for exosome separation.

Ultracentrifugation (UC) is currently the gold-standard in exosome isolation and widely used in laboratories,^136^ where roughly 56% of all exosome isolation is performed using this technique.^150^ UC is based on the difference in the sedimentation rate of particles, which is affected by their size, density, and shape.^151^ By applying centrifugal forces, sample components can be separated sequentially according to their physical properties, and the density and viscosity of the solvent. Given the same particle density, larger particles sediment faster than smaller ones; therefore, smaller particles, such as exosomes, can be isolated with a series of sequentially increasing rotational speeds. Typically, cells, dead cells, and cell debris are first removed as pellets at a lower speed (300×g to 1000×g), and the supernatant is carefully aspirated and used for the next round of centrifugation. Finally, ultracentrifugation is carried out at 100 000×g for 70 min to obtain the pellet of exosomes.^136^

The density gradient for exosome isolation is another popular method in which the medium is modified to increase the density of the solution from top to bottom. A sample of bioparticles is also added to the surface of the medium to create a narrow layer. Followed by ultracentrifugation, these bioparticles move towards the bottom of each density gradient layer to create discrete solute zones^59^ where exosomes can be separated, and finally collected by fraction collection.

Despite their popularity, both of the above methods present a few drawbacks: they are extremely time-consuming (normally lasting between 5 and 10 h or more^152–155^), have low recovery rates [ranging between 5% and 25% (Ref. 156)], and low yield (375 *μ*g of protein from 6 × 10^8^ cells approximately).^149,157,158^ These last two factors lead to the need for great sample volumes for isolation. Furthermore, they may cause a change in the exosome's morphology and composition due to high centrifugal forces.^159–161^

#### 2. By size

Purification of exosomes can be performed based on the size differences of various particles.^162^ Ultrafiltration (UF) and size exclusion chromatography (SEC) are two main techniques that have been used for size-based isolation of exosomes. Ultrafiltration is often combined with UC, replacing a few of lower speed rounds of spinning in the UC method with filtration.^157^ A Nano-porous membrane with the typical pore size ranging between 0.1 and 0.001 *μ*m (Refs. 157 and 163) is used to filter a suspension of bioparticles which are sorted out based on their sizes [Fig. 3(c)].^59^ Following this step, the standard UC protocol continues. Compared to the UC method, ultrafiltration has shown a higher purity (5-folds higher compare to UC) of isolated exosomes as well as less time consumption (1–2 h).^164,165^ Nonetheless, trapping bioparticles in nanopores can cause clogging issues which result in low recovery rates (e.g., only small amounts of exosomal proteins, AQP2 and TSG101, could be recovered due to the adhesion to the nanomembrane).^166^ Furthermore, due to the high forces applied to bioparticles as they pass through nanopores, high shear stresses can be generated which may change the morphology of EVs or even cause lysis.

Size exclusion chromatography (SEC) is another size-based purification technique that is also known as “gel filtration.”^167^ The working principle of SEC is based on the hydrodynamic radii of bioparticles.^168,169^ Its primary phase or stationary phase contains spherical porous particles with a specific pore size. Depending on the size difference between pores and bioparticles, some bioparticles will be retained according to their diffusion [Fig. 3(d)]. In this way, bioparticles with large hydrodynamic radii like exosomes cannot pass through these pores and are excluded. In a recent study, Baranyai *et al.* compare both the UC and SEC method for exosome purification.^140^ They claimed that unlike UC, SEC is a promising method for exosome purification without albumin presence in purified exosomes. Furthermore, they highlighted that only a small portion of exosomes (1%–5%) can be isolated from the blood with SEC or UC, which demonstrates the low capability of both SEC and UC for exosome isolation.

#### 3. By function

Aside from density and size-based separation techniques, exosome isolation can be performed based on the functionality of biomolecules. Immunoaffinity and polymer precipitation are two types of exosome purification that work based on their functionality.^59^ In these methods, chemical properties of bioparticles' surfaces play vital roles in the separation process.

One of the conventional function-based exosome purification methods is immunological-based separation [Fig. 3(e)]. Each type of EV has specific proteins on its surface which interact with their specific antibodies, peptides or polysaccharides. Thus, exosomes can be pulled down from other components of the sample.^151,157,170^ CD9, CD41, CD63, and CD81 are common standard exosomal surface markers for immunoaffinity-based isolation. Exosomes can be isolated by immobilizing these antibodies on various surfaces such as magnetic beads, plates, chromatography matrices, and microfluidic platforms.^90,146,171^ In the case of immunoaffinity magnetic beads, Zarovni *et al.*^150^ found that antibody-coated magnetic particles can provide a similar efficiency as UC by using a small amount of the cell culture supernatant (0.1 ml). In this method, an external force displaces the bonded magnetic beads and bioparticles to the area of interest. Nonetheless, they found that even higher yields (10–15 times higher than UC) can be obtained using the plasma sample instead of the cell culture supernatant. Unlike magnetic beads, immunoaffinity chromatography immobilize antibodies or antibody-related agents in the “stationary phase”; while the sample or “mobile phase” passes through. According to the affinity of the sample towards the immobilized antibodies, elution times differ. This will allow the target sample to bind to the stationary phase long enough for the rest of the sample components to elute first. After this, targeted molecules can be eluted and collected for further analysis with the use of other techniques like mass spectrometry (MS).^172^ The similar principle has been applied to plate-mounted immunoaffinity; however, instead of columns, wells have been used where every well in the plate was coated with an antibody.^173^ In another study, Tauro *et al.*^149^ reported that immunoaffinity isolation provides a higher purity (about 3 times higher amounts of the normalized spectral count ratios (N_sc_) for various surface protein biomarkers) compared to both differential UC and density gradient methods. The main drawback of this method is that large quantities of biological samples cannot be processed.^174^ Instead, only pre-concentrated small volumes are suitable for this method.^136,157^ Moreover, besides exosomes, other types of EVs (such as microvesicles and apoptotic bodies, proteins, cells, and cell debris) may present similar proteins to exosomes, resulting in low purity.^151^

Another method, precipitation, is achieved when the solubility or dispersibility of molecular components, such as exosomes, can be changed using volume-excluding polymers [Fig. 3(b)].^171^ This principle is based on the effective volume of a solution being inaccessible in the presence of polymers in the precipitating reagent. By binding with water molecules, these polymers saturate the solution forcing less soluble components such as exosomes to precipitate. Once precipitated, these target molecules can be sedimented for further isolation steps through low-speed centrifugation (1500 × g) or filtration.^171,175^ One of the most widely used polymers for this purpose is polyethylene glycol (PEG). This polymer allows an effective isolation of exosomes since it provides an easy and low-cost (no need for special equipment) precipitation procedure from a cell culture supernatant.^175^ High-resolution electron microscopes has been used to visualize the size and morphology of isolated exosome aggregates, verifying the mechanism of PEG-based precipitation.^175^ Commercial exosome precipitation kits are widely available in the market and are advertised to be compatible with various body fluids such as breast milk.^171,176^ However, the main drawback of this method is the co-precipitation of non-exosomal components such as proteins and polymeric materials, which leads to a lower efficiency of exosome isolation.^150^

### B. Emerging—Isolation platforms

In recent years, the field of microfluidics has allowed the development of novel methods for exosome purification.^157^ Microfluidics provides platforms, including micron-sized channels, for processing small amounts of fluids (microliter to picoliter).^177,178^ Most of the microfluidic devices are fabricated with a specific polymer called poly (dimethylsiloxane) or PDMS.^177^ PDMS is optically transparent and biocompatible; these properties make PDMS a useful material in bio-microfluidics device fabrication.^179^ Microfluidics devices can have different components based on their applications as well as the approach of separation. These components include microchannels, connecting tubes, microvalves, micromixers, and micropumps.^177,180^ By allowing the manipulation and processing of small amounts of fluids through microscale channels,^177^ it has been shown that microfluidic platforms can sort exosomes with a high level of purity and sensitivity while reducing the cost, the volume of reagents consumed, and time invested in the procedure.^145–148,181^ Generally, microfluidic-based methods are classified into two main groups: active and passive. The first methods are defined by the exertion of external forces, while the second ones rely only on the use of hydrodynamic and surface forces.^182^ Table III exemplifies different techniques according to this classification.

**TABLE III.** Summary of microfluidics platforms based on active and passive methods of isolation.

Microfluidics platforms	
Active	Passive
Acoustophoresis	On-chip centrifugation
Electrophoresis-driven filtration	Inertial lift force
Dielectrophoresis	Viscoelastic flow
Magnetophoresis	Filtration
	Immunoaffinity

Following the categories used for conventional methods (density, size, and function), Table IV shows different examples of microfluidic-based methods with their respective throughput, recovery yield, isolation capacity, and input sample. Techniques such as acoustophoresis, filtration, and viscoelastic flow have the highest recovery yield, which is one of the most important factors when evaluating the efficiency of isolation. Meanwhile, methods like pressure-driven, electrophoresis-driven filtrations, and nanowire trapping have the lowest recovery rates. In Secs. V B 1, we discuss some of these methods in detail.

**TABLE IV.** Microfluidics-based exosome isolation techniques.

Isolation method	Exexosome isolation approach	Input sample	Throughput (*μ*l/min)	Isolation capacity (*μ*l)	Recovery yield (%)	References
By size	Acoustophoresis					
	1. Purification of extracellular microvesicles	Packed red blood cell (pRBC) units	0.24	10	80	183
	2. Isolation of exosomes from whole blood	Undiluted whole blood	4	1500	82	138
	Electrophoresis-driven filtration	Whole blood	2	240	1.5	184
	1. Integrated centrifugal microfluidic platform (Exodisc)	Urine	36	1000	95	185
	2. Doubled filtration	Urine	33	8000	74.2	186
	3. Nanowire trapping	BSA and liposomes	10	100	10	187
	Inertial lift force	Blood	70	NA	NA	188
	Viscosity flow	Serum	10	100	93.6	189
By density	On-chip centrifugation	Cell culture media	N/A	10	N/A	190
By function	Immunoaffinity					
	Integrated immunoisolation and protein analysis of circulating exosomes using microfluidic technology	Plasma	2	30	N/A	191

#### 1. Isolation by density (on-chip centrifugation)

In 2018, Yeo *et al.*^190^ introduced a label-free extraction of EVs by coupling microfluidic designs with centrifugal nanoparticle separation and extraction [Fig. 3(f)]. Their micro-chip, called μCENSE, used centrifugal micro-hydrodynamics as the isolation method because they do not require external elements (syringe pumps or others) to introduce the samples into the microchannels. The microfluidic chip was divided into three parts: (1) a serpentine inlet channel (to have sufficient hydrodynamic resistance towards fluid movement), (2) a microfluidic separation channel, and (3) two outlets.

As previously mentioned, this method is based on centrifugal forces where larger particles will migrate longer distances in comparison to smaller ones due to the force being proportional to the square of the particle diameter. Particles inside the separation channel experience different forces such as centrifugal, coriolis, buoyancy, and hydrodynamic drag, however, at the steady-state, the centrifugal force is equal to the sum of all other forces. Therefore, by rotating the motor assembly, the whole micro-chip rotates and generates these forces inside the microchannels.

#### 2. Isolation by size

##### a. Acoustophoretic isolation

In this method, forces generated by acoustic waves are used to manipulate or isolate targeted molecules [Fig. 3(g)]. Generally, two techniques are used to create acoustic waves: Bulk Acoustic Wave (BAW) and Surface Acoustic Wave (SAW). BAW uses a resonator, oscillating at a specific frequency, and vibrates the transducer as a bulk. In contrast, SAW generates the acoustic pressure field by applying voltage to interdigital transducers (IDTs) patterned on the surface of a piezoelectric material, which generates the displacement. The SAW technique can be further classified into three categories: Traveling Surface Acoustic Wave (TSAW); Standing Surface Acoustic Wave (SSAW); and Pseudo Surface Acoustic Wave (PSAW); depending on certain parameters such as number and location of IDTs, frequencies, and amplitude of acoustic forces.^182,192^ The range of frequencies used for any of these acoustophoretic methods (MHz) is similar to ultrasound imaging and does not distort the cellular properties, and hence, is biocompatible.^138^ However, the main drawback for this isolation method is the challenging and time-consuming fabrication process where highly precise alignments are required. Although this method mainly depends on the particle size, the density, and compressibility differences between particles and the fluid medium are also important factors.^193^

For exosome isolation from a whole unprocessed and undiluted blood sample, Wu *et al.*^138^ used the SSAW technique. Their device consisted of two sequential SAW modules. The first cell-removal module separated larger components of blood such as red blood cells (RBCs), white blood cells (WBCs), and platelets (PLTs). Once a cell-free plasma was obtained from the first module, the next module (i.e., exosome-isolation) separated the nanoscale components such as microvesicles, apoptotic bodies, and exosomes.

##### b. Filtration

Filtration methods have widely been used to separate and isolate components of biological samples, as it is a label-free process without the need for external forces. In this case, nano-filters, nano-porous membranes, or nanoarrays^160^ are usually used in microchannels to separate particles based on their size [Fig. 3(h)]. Woo *et al.* presented an integrated microfluidic platform called Exodisc for label-free exosome purification.^185^ The device is based on a nanofiltration and centrifugation process. When the microchip is spun at a relatively low acceleration (<500×g), the biological sample passes through different nano-filters with a pore size ranging from 600 to 30 nm, allowing the concentration of EVs. Compared to UC methods, this device has a total recovery rate of 95% and the complete process can be performed within 30 min. In summary, larger particles such as dust are separated from the input sample by spinning the disk at 3000 rpm. After that, the sample is transferred to the first chamber and a second valve is opened to let the sample pass through 2 nano-filters (I and II). Filter I with a pore size of 600 nm captures large particles, while filter II of 20 nm only allows small particles to pass through. Therefore, EVs are retained before filter II and collected by a washing buffer introduced through the microchannels. This transferring is carried out by reversible diaphragm valves.

Another passive method of EV isolation was introduced by Villarroya-Bltri *et al.*,^32^ which trapped exosomes using ciliated micropillars. These posts, set perpendicular to sample flow, were covered in small protrusions with nanosized gaps that acted as a filter for the sample. In essence, large cells could not enter the micro-pillared region, so they were collected at the first row of pillars, whereas smaller molecules, such as proteins, could pass through the micropillar area without being caught in the nanowires. Exosome-like vesicles were trapped by the nanowires. As with any label free method, the main drawback of this filtration technique is lack of specificity. Anything smaller than the space between the posts and larger than the space between the wire protrusions has the possibility of being caught. As such, a wide variety of cellular products may be isolated. Also, the setup could potentially cause cell lysis and accumulation of the resulting debris on the nanowires. This would result in clogging of the device and preventing it from capturing target molecules.

##### c. Inertial lift force

One of the more straightforward passive methods to separate cells in microfluidics platforms is through the use of inertial lift forces. These forces can be used to displace the particles laterally across the microchannels with a sufficient flow rate and velocity differences between particles and fluid.^194^ Based on this principle, Dudani *et al.*^188^ proposed a rapid inertial solution exchange (RInSE) method, utilizing inertial lift forces to move microparticles across the channel. However, since exosomes are nano-scaled, inertial lift forces cannot influence them significantly. Therefore, exosomes are incubated with beads to create a larger exosome-bead complex [similar to Fig. 3(j)]. As the inertial lift force is proportional to $a^{6}$ (where a is the diameter of particles), acellular contaminants (a < 5 μm) do not experience a considerable inertial lift force and remain inside the walls of the channel while the exosome-bead complex is influenced by the inertial lift force (on the order of 2 nN) and moved toward the buffer. Therefore, the isolated exosomes are collected from the collection outlet. Since non-exosomal EVs are not large enough, they are not influenced by the inertial lift forces and do not move towards the collection outlet. Dudani *et al.* achieved significant inertial lift forces with a high aspect ratio channel (a width of 100 *μ*m and a height of 30 *μ*m) to decrease the shear gradient in the center of the channel.

##### d. Viscoelastic flow

Another passive method for cell sorting is through viscoelastic microfluidics, where elastic lift forces are exerted by a viscoelastic medium to the particles [Fig. 3(i)].^189^ To create the viscoelastic medium, different polymers, such as diluted (low concentration 0.1% w/w) poly-oxyethylene (PEO), can be used. The PEO polymer makes the fluid highly viscoelastic and causes an imbalance in the first normal stress difference across the microchannel. This imbalance creates an elastic force proportional to the volume of particles. As a result, bioparticles can be positioned laterally across the width of the microchannel based on their volume as the elastic lift force is proportional to $a^{3}$ (where a is the diameter of bioparticles).The main advantage of this method (compared to the inertial lift force) is that beads (for size amplification) are not required as the elastic lift force is about one order of magnitude stronger (∼1 pN) than the inertial lift force (∼0.1 pN).

#### 3. Isolation by function

As previously discussed, immuno-affinity capturing is widely used as a conventional exosome isolation method, and it can be integrated into microfluidic platforms [Fig. 3(j)]. From the microfluidics point of view, there are two types of immuno-affinity-based isolation involving: (1) modification of the surface of microchannels with antibodies and (2) the use of affinity particles or magnetic beads. An example of the latter is the promising study performed by He *et al.*^191^ who integrated magnetic beads with antigens for exosome capture/isolation. These surface biomarkers allowed the manipulation of exosomes with an external magnetic field. Antibody-labelled magnetic beads were pre-mixed with the plasma sample in the chip and sedimented inside a micro chamber by an external magnetic source. A lysis buffer was then injected into the chip to release intra-vesicular proteins of captured exosomes for further analysis. The released intra-vesicular proteins were bound to another set of antibody-labeled magnetic beads and were retained in the second micro chamber by applying the external magnetic force. To achieve a sandwich immunodetection, chemifluorescence reagents were added to the solution, and in this way, specific protein biomarkers were detected.

In summary, microfluidic-based exosome separation methods are a promising alternative to current gold standard conventional methods. However, one of the main drawbacks of microfluidics platforms is their complicated fabrication (the need for a cleanroom and intricacies of photolithography). Both physical and chemical properties of exosomes can be used in microfluidic-based separation methods. Among all the currently available methods, immunoaffinity-based separation has attracted more attention as it is simpler in both operation and fabrication. Nonetheless, the high dependency of this method on specific antibodies of each target of interest is a major challenge. Acoustic-based separation of exosomes has shown to be a promising approach for exosome isolation as it is biocompatible and does not change the morphology of exosomes after separation. However, complex fabrication processes are the main demerits of this method. Furthermore, numerous microfluidics devices have been introduced which work based on the size and density (such as filtration, on-chip centrifugation, respectively). Although these devices do not require any additional reagents (such as antibodies), the size overlap and chance of clogging are two main issues of these devices. All in all, microfluidics-based exosome isolation is the emerging alternative method with a high sensitivity, high recovery rate, and low required volume of input samples. These features can be implemented in clinical applications for personalized medicine and as personal pre-diagnostic devices in the future.

## VI. CHARACTERIZATION

A thorough analysis of exosome characteristics is often challenged by the heterogeneity of EV isolates, resulting in a mixed size distribution, difficulties in profiling cargo contents, and microscopy. Generally, characterisation of the exosome has focused around their size, morphology, proteomic, lipidomics, and genomics. Subsections VI A–VI C will describe the established and emerging techniques in exosome characterization.

### A. Size and shape

Currently, the gold standards in morphology characterization are Electron Microscopy technologies. Formerly, exosomes were frequently described as having a cup-shaped morphology.^195^ This is observed when using Transmission electron microscopy (EM) techniques, and it is now generally assumed to be wrong due to conflicting data from Scanning EM techniques which indicate that exosomes are roughly spherical with a consistent size distribution.^196^ TEM works by directing a wide electron beam through a thin sample and then spreading the beam with a lens to produce an image. The contrast of the image is produced as a result of electron scattering as the beam crosses the sample. Unfortunately, this technique requires a very thin sample, and hence a significant effort is required for sample preparation which may affect sample properties such as morphology. SEM, on the other hand, bounces a very thin stream of electrons off a sample and compiles a three-dimensional image from the resultant electronic signals: much less sample preparation is needed. EM techniques are attractive due to their ability to obtain a resolution of 0.1 nm and 3 nm for TEM and SEM respectively.^197^ Thus, the exosome of 40 nm can be clearly defined. Despite these advantages, exosome populations must be isolated and fixed prior to imaging which may alter the characteristics of the exosome and greatly limits this technique to static visualisation of the size and shape only with questionable accuracy. Though exosomes are commonly described as having diameters ranging from 40 to 100 nm, Wu and colleagues characterised exosomes from B16F0 cells, isolated by conventional ultracentrifugation methods, to range from 139 to 185 nm.^196^ This, along with the observation that exosomes appear to shrink over time,^198^ suggests that researchers should report the length of time between isolation and characterisation.

Another common technique for size determination is Nanoparticle Tracking Analysis (NTA). This piece of software uses a video file, obtained from any microscopic technique capable of observing the movement of exosomes, to determine the size and concentration of the particle. It works by tracking individual particles' velocity and Brownian motion frame by frame. As displacement of a particle in a solvent is related to certain parameters (temperature, solvent viscosity, and size of the particle) the Stokes-Einstein equation can be used to calculate the size if displacement, temperature, and viscosity are known.^199^ The detection limit of NTA is dependent on the ability to see the particle and thus the resolving power of the microscope. As it can track multiple particles simultaneously, NTA is able to characterize polydispersed samples.^200,201^ This method is similar to Dynamic Light Scattering (DLS) techniques which calculate the hydrodynamic radii of particles based on the fluctuations in laser transmission caused by the Brownian movement of the particles. While DLS can characterise particles between 1 nm and 6 μ m, it is only accurate with particles of a homogenous sample, and it is easily influenced by the existence of larger particles in the sample which makes NTA a more versatile and reliable method.^202,203^

### B. Molecular profiling

There has been a prodigious number of studies focusing on EVs molecular profiling over the past decade, including proteomics, lipidomics, and genomics. Exosome cargo contents retain information of the cell origin, and hence detailed molecular profiling could not only reveal exosome functions, but also provide clues in exosome biogenesis and identifying potential EV biomarkers for diseases detection/diagnosis. As mentioned earlier, systematic profiling of homogenous EVs with a specific subpopulation has not been accomplished, resulting in contamination of other types of EVs or EVs from different cell phenotypes. As a result, prior to molecular profiling, EVs need to be isolated using standard isolation/purification protocols and the presence of exosomes needs to be confirmed by methods such as western blot, TEM or DLS. As a matter of fact, the success of EV molecular profiling heavily relies on the isolation and separation process.

#### 1. Proteomics and lipidomics

Various standard proteomics approaches have been applied in exosome proteomic analysis. In particular, liquid chromatography coupled tandem mass spectroscopy (LC-MS/MS) and two-dimensional gel electrophoresis (2DGE) are predominantly used.^53,204–206^ Mass spectrometry (MS) based proteomics provide comprehensive analysis of exosome protein contents. Briefly, after standard EV purification, proteins are extracted from the EV lysate, and made to undergo peptide preparation. Peptide fragments are required for MS analysis since they are more easily fractionated by LC, ionized and fragmented by MS, which results in more accurate measurements.^205,207^ Most studies in EV research use in-solution digestion to yield peptides, which include reduction, alkylation, and tryptic digestion.^206,208^ The peptides will then be separated by one or more steps of high-pressure LC to be separated into several components. Finally, the peptides enter the tandem MS (MS/MS), where two stages of MS are performed. In the first stage, ions are formed in the ion source and separated by their mass-to-charge ratio. The second MS analyser then selects the ions of interests and fragments them for further separation and detection. The peaks are processed and matched against the database to reveal the protein identity.^207^ The MS-based proteomics allows thousands of proteins to be identified and quantified from complex samples.

2DGE is a traditional and one of the most widely used technique to study the proteome of a cell.^204,209^ In 2DGE, proteins are first separated by their isoelectric points (where the protein has a neutral charge) and subsequently by mass, thus distributed in a two-dimensional gel.^209^ During the first step, proteins move along the gel under a pH gradient, accumulating at their isoelectric point. These proteins are then treated with sodium dodecyl sulfate (SDS) along with reducing reagents to unfold into a linear structure with a negative charge from the SDS molecules. Finally, in the second dimension, an electric potential is applied at the 90-degree angle from the first field and the proteins are separated on the gel according to their molecular mass. Each spot on the resulting two-dimensional array corresponds to a single protein species in the sample and the spots of interests are further analysed using MS-based techniques.

Compared to proteomics, studies on lipid composition and the presence of metabolites in EVs are underrepresented. Lipid species consists permutations of the head and tail groups, with various possible modifications and configurations, which results in heterogeneity and complications in analysis. Most lipidomic in EV research have been performed using the MS-based method, and the techniques and workflow used in lipidomics are similar to proteomics.^210^ Briefly, lipids are extracted through the EV lysate in liquid-liquid extraction using organic solvents, then separated and analysed by gas chromatography-mass spectroscopy (GC-MS) or LC-MS.^205,207^

Using these techniques, more than a thousand different proteins may be isolated from 25-ml of urine.^211^ Generally, TSG 101, annexins, tetraspanins, and ESCRT proteins and associated proteins (such as ALIX) are identified.^212^ However, standard exosomal markers such as major histocompatibility complexes, Flotillin, Hsp70s, and tetatrspanins CD9; CD63; and CD81, among other often identified proteins are found in other EV populations.^213^

The main limitation in EV proteomics and lipidomics are the contamination of other types of EVs, thus the accuracy largely depends on the isolation techniques. For this reason, it is currently impossible to separate EVs based on their biogenic origins, which in turn makes the analysis of bona fide exosomes challenging. Nevertheless, advances in molecular profiling would help identify useful biomarkers for EV isolation, which in turn will advance the field tremendously.

#### 2. Genomics

Determining the RNA content of the exosome has the same challenges as determining the proteomic content does. While degradable in biological fluids, RNA exists free of cells and other membrane compartments (such as EVs), it nonetheless exists in biological fluids including blood and urine.^214,215^ Even so, the literature notes a few things about RNA in exosomes. In terms of noncoding RNAs, 3′ uridylated strands are highly enriched in exosomes while their 3′ adenylated isoforms are not.^216^ mRNA, ncRNA, miRNA, snRNA, tRNA, rRNA, piRNA, and mtRNA, among others, have been isolated from exosome samples.^217,218^ Currently, commercial kits appear to be the common method of RNA extraction and isolation.^196,219,220^ Though reverse-transcription-quantitative polymerase chain reaction (RT-qPCR) is the main method for profiling;^221^ this works by converting the isolated RNA into cDNA and amplifying it via a PCR.^222^ From here, the strands of DNA can be profiled for length and nucleotide sequences.

### C. Microscopy and nanoscopy for exosome imaging

While *ex vitro* methods such as genomic assays, TEM and DLS provide essential information of sizes and contents, their accuracies are limited by the absence of standard isolation protocols. Moreover, established characterization methods failed to visualize exosomes in their natural habitat nor are they able to capture the dynamic process *in vitro*. Recent advances in fluorescent microscopy (FM) provided non-invasive, direct approaches to image exosomes *in vitro* and *ex vitro.* Additionally, by tracking fluorescently labelled exosome biomarkers, the dynamics of exosome biogenesis, release and cellular uptake can be revealed. Furthermore, FM allows more than two fluorescent dyes to simultaneously stain and label different compartments of the cells.^223^ This is particularly useful in detecting exosome populations without solely relying on the isolation quality since the colocalization of several exosome specific proteins would more likely to confirm the presence of this subset of EVs. In general, exosomes are visualized either by directly labelling certain membranes/proteins using fluorescent dyes or through fluorescent fusion proteins that were introduced to the host cell cytoplasm via transfection. Labelling with fluorescent dyes is convenient and can be adjusted easily. However, direct labelling often suffers from false positive results by excess and free dyes. Fluorescent proteins offer stable fluorescent signals and the labelling is more precise, however, the technique is lengthy. Once they are labelled, these tags can also be used for surface protein characterization and affinity based purification.^224,225^

#### 1. Direct post-isolation labelling for imaging

Exosomes can be labelled with either lipophilic dyes or antibody-conjugated dyes. To do this, exosomes are required to be separated and purified from cell cultures, following incubation with specific fluorescent dyes. Lipophilic dyes such as PKH67, Dil, DiD or DiR embed in the lipid bilayer of the exosomes, and they have been widely used.^226–229^ In these studies, lipophilic dyes label all membrane structures, hence not specifically to exosomes.

Immunofluorescence (IF) targets surface proteins found on exosomes via fluorescently labelled antibodies. There are two ways of IF labelling: direct or indirect. Direct IF uses a fluorescent primary antibody to tag exosomes, while indirect IF uses a primary antibody to target exosomes and fluorescent secondary antibodies for visualization. Currently, indirect IF is the preferred method in detecting endogenous proteins involved in exosomes due to greater flexibility in the labelling strategy. For example, Kibria *et al.* treated MDA-MB-231 derived exosomes with mouse anti-human CD63 antibodies then incubated with Alexa 488 conjugated goat anti-mouse IgG secondary antibodies.^230^ The treated exosomes were observed under a confocal microscope to show the CD63 expression level. Similar approaches, often coupled with lipophilic dyes, were used in other studies to visualize exosome uptake as well as internalization and locations in recipient cells.^227,231^

Although widely used, post isolation labelling techniques have a few limitations: free label or non-specific absorption exist even after multiple washing steps, giving false-positive signals and lower signal-to-noise ratio. Additionally, prior to labelling, exosomes need to be purified from conditioned media or body fluids. Given the current challenges in exosome isolation, this method is unreliable for exosome labelling.

#### 2. Genetic tagging by fluorescent proteins

Due to the limitations of the post-production IF labelling techniques, many studies have explored options of using fluorescent proteins. Plasmids are engineered to express exosomal surface proteins fused with fluorescent protein tags (e.g., EGFP, mCherry). They are then transfected in host cells where the tagged proteins have the chance to be incorporated into exosomes.^232^ For example, to elucidate the different subcellular distributions of Rab 27a and Rab 27 b proteins, HeLa cells were simultaneously transfected with mCherry-Rab27a or GFP-Rab27b, then stained with anti-CD63.^48^ The colocalization of the fluorescent signals revealed Rab 27a and Rab 27b functions in different compartments, and they perform non-redundant tasks in the exosomal pathway. Lai *et al.* developed a fluorescent EV labelling strategy to achieve live-cell imaging of EV release, uptake, and exchange between different cell populations, as well as microscopic quantification and flow cytometry analysis.^232^ For the generation of fluorescent EV reporters, a palmitoylation signal is generically fused in-frame to the N-terminus of the enhanced green fluorescent protein and tandem dinmer Tomato, generating PalmGFP, and Palmtd Tomato.

Fluorescent proteins have their own limitations such as having little control over the brightness once its transfected, dimmer signals, and lower photo-physical properties compared to organic dyes.^233^ Additionally, precise tagging of exosomes requires exclusive surface proteins to exosomes, that are yet to be discovered. Therefore, most studies that use fluorescent protein labelling would still go through post-isolation process and focus on the exosome uptake process. More importantly, such transfection can potentially alter other cellular pathways, although it cannot be easily applied in clinical settings due to the long-lasting transfection and potential adverse effect on host cells.

#### 2. Advanced fluorescent microscopy technique

Conventional FM has difficulties in resolving objects smaller than half of the wavelength of light used for imaging due to the limitations of light diffraction.^234^ This phenomenon, also referred to as the resolution limit, is usually around 200 nm and is a major obstacle in optical microscopy.^235,236^ Due to the small size of exosomes, it is challenging to obtain clear images to differentiate exosomes from other cell structures.^237^ Usually, combinations of characterization techniques are required to validate the fluorescent imaging results. However, it is still impossible to provide clear and direct insights on exosome localization and dynamic movements. Recent progress in super-resolution imaging has provided novel tools in exosome characterization, especially with total internal reflection fluorescence microscopy (TIRF) and single-molecule localization microscopy (SMLM) including photoactivation localization microscopy (PALM) and stochastic optical reconstruction microscopy (STORM).^234,236–238^ Particularly, TIRF has become a dominant technology for visualizing the cell plasma membrane. TIRF exploits the evanescent wavelength that is induced when light is totally internally reflected at the glass-water interface. The evanescent wavelength propagates parallel to the glass-water interface and decays exponentially perpendicular in direction from this interface, thus penetrating sample into about 100 nm. Usually a fluorophore is on the surface of the interface, which can be excited by the evanescent field and only 100 nm of thickness would be excited giving out a reduced signal-to-noise ratio. This technique is well used in studies of the plasma membrane, as well as endocytosis and exocytosis processes.^235^ On the other hand, SMLM techniques are based on the ability to image subsets of fluorophores at a time, switching between fluorescent and non-fluorescent. As a result, non-overlapping single fluorophores can be imaged. Particularly, direct STORM uses synthetic photoswitchable dyes such as cy5 and Alexa Fluor 647 as probes to achieve the on-off fluorescent cycles.^239,240^ PALM uses photoactivatable fluorescent proteins that can be activated on and off with specific illumination.^241^ The recorded diffraction limited images (characterized by the point spread function) were then fitted with two dimensional Gaussian functions to determine the centroid of each molecule. Subsequently, all the subsets of frames are then used to reconstruct a final super resolution image of the sample, built point by point.^237^

Chen *et al.* investigated the power of super resolution in exosome imaging using both PALM and TIRF techniques. In their study, cancer-derived exosomes were isolated and the membrane receptors CD63 and HER2 were labeled with photoswitchable probes Alexa Flour 488 and 647 through indirect IF.^227^ Alexa Fluor dyes were chosen due to their good photoswitching properties and high photon yield. In addition, the exosome membranes were labelled with CM-Dil dyes to confirm the exosome existence. The TIRF technique was used to visualize and locate the exosome membrane, then PALM/STORM methods were used to image the Alexa Fluor dyes on the exosome receptors. Using PALM/STORM method, the final reconstructed super-resolution image of exosomes achieved a spatial resolution at 70 nm. Super-resolution imaging was also conducted to visualize exosome internalization, and the colocalization of exosomes and lysosomes revealed large portions of exosomes were transported to lysosomes for further degradation. This study provided evidence of using super-resolution techniques to visualize exosomes.

## VII. CONCLUSIONS

With substantial research being dedicated to exosome applications, it is vital to understand the progress made and the persisting challenges. Although EVs analyses have impressively evolved in the last decades, the exact mechanisms of biogenesis are still unknown. Conversely, improvements in the methods of exosome isolation and purifications are needed in order to study the cargo contents and functions, which would shed light on the biogenesis in return. Once such limitations are overcome, new biomarkers can be identified for exosome characterization and use them in diagnostic applications. Moreover, with more information on exosome biogenesis and functions, there would be significant opportunities to manipulate their composition, properties, and cell interactions to further advance their therapeutic application. Nonetheless, recent advances in using exosomes as biomarkers for disease detection and as natural drug/gene delivery systems have been stimulating. The potential of using exosomes as a therapeutic platform is clearly demonstrated.

In conclusion, developing efficient and reliable isolation methods is urgent to further advance in this field. To fully utilize the potentials of the potentials, basic research and emerging new technologies need to be integrated, which will set the foundations for their therapeutic applications.

## References

[1] M. Colombo , G. Raposo , and C. Théry , “ Biogenesis, secretion, and intercellular interactions of exosomes and other extracellular vesicles,” Annu. Rev. Cell Dev. Biol. 30, 255–289 (2014). 10.1146/annurev-cellbio-101512-122326 25288114

[2] N. Yim *et al.*, “ Exosome engineering for efficient intracellular delivery of soluble proteins using optically reversible protein–protein interaction module,” Nat. Commun. 7, 12277 (2016). 10.1038/ncomms12277 27447450 PMC4961865

[3] I. L. Colao , R. Corteling , D. Bracewell , and I. Wall , “ Manufacturing exosomes: A promising therapeutic platform,” Trends Mol. Med. 24, 242–256 (2018). 10.1016/j.molmed.2018.01.006 29449149

[4] G. van Niel , G. D'Angelo , and G. Raposo , “ Shedding light on the cell biology of extracellular vesicles,” Nat. Rev. Mol. Cell Biol. 19, 213–228 (2018). 10.1038/nrm.2017.125 29339798

[5] S. L. N. Maas , X. O. Breakefield , and A. M. Weaver , “ Extracellular vesicles: Unique intercellular delivery vehicles,” Trends Cell Biol. 27, 172–188 (2017). 10.1016/j.tcb.2016.11.003 27979573 PMC5318253

[6] G. Raposo and W. Stoorvogel , “ Extracellular vesicles: Exosomes, microvesicles, and friends,” J. Cell Biol. 200, 373–383 (2013). 10.1083/jcb.201211138 23420871 PMC3575529

[7] E. Cocucci and J. Meldolesi , “ Ectosomes and exosomes: Shedding the confusion between extracellular vesicles,” Trends Cell Biol. 25, 364–372 (2015). 10.1016/j.tcb.2015.01.004 25683921

[8] M. Tkach and C. Théry , “ Communication by extracellular vesicles: Where we are and where we need to go,” Cell 164, 1226–1232 (2016). 10.1016/j.cell.2016.01.043 26967288

[9] A. Waldenström and G. Ronquist , “ Role of exosomes in myocardial remodeling,” Circ. Res. 114, 315–324 (2014). 10.1161/CIRCRESAHA.114.300584 24436427

[10] R. Kalluri , “ The biology and function of exosomes in cancer,” J. Clin. Invest. 126, 1208–1215 (2016). 10.1172/JCI81135 27035812 PMC4811149

[11] J. Huotari and A. Helenius , “ Endosome maturation,” EMBO J. 30, 3481–3500 (2011). 10.1038/emboj.2011.286 21878991 PMC3181477

[12] X. Yu , S. L. Harris , and A. J. Levine , “ The regulation of exosome secretion: A novel function of the p53 protein,” Cancer Res. 66, 4795–4801 (2006). 10.1158/0008-5472.CAN-05-4579 16651434

[13] R. Kojima *et al.*, “ Designer exosomes produced by implanted cells intracerebrally deliver therapeutic cargo for parkinson's disease treatment,” Nat. Commun. 9, 1305 (2018). 10.1038/s41467-018-03733-8 29610454 PMC5880805

[14] M. Colombo *et al.*, “ Analysis of ESCRT functions in exosome biogenesis, composition and secretion highlights the heterogeneity of extracellular vesicles,” J. Cell Sci. 126, 5553–5565 (2013). 10.1242/jcs.128868 24105262

[15] W. M. Henne , N. J. Buchkovich , and S. D. Emr , “ The ESCRT pathway,” Dev. Cell 21, 77–91 (2011). 10.1016/j.devcel.2011.05.015 21763610

[16] O. Schidt and D. Teis , “ The ESCRT machinery,” Curr. Biol. 22, R116–R120 (2012). 10.1016/j.cub.2012.01.028 22361144 PMC3314914

[17] J. R. Mayers *et al.*, “ ESCRT-0 assembles as a heterotetramic complex on membranes and binds multiple ubiquitinylated cargoes simultaneously,” J. Biol. Chem. 286, 9636–9645 (2011). 10.1074/jbc.M110.185363 21193406 PMC3058970

[18] K. Trajkovic *et al.*, “ Ceramide triggers budding of exosome vesicles into multivesicular endosomes,” Science 319, 1244–1247 (2008). 10.1126/science.1153124 18309083

[19] D. J. Gill *et al.*, “ Structural insight into the ESCRT-I/-II link and its role in MVB trafficking,” EMBO J. 26, 600–612 (2007). 10.1038/sj.emboj.7601501 17215868 PMC1783442

[20] T. Wollert and J. H. Hurley , “ Molecular mechanism of multivesicular body biogenesis by ESCRT complexes,” Nature 464, 864–869 (2010). 10.1038/nature08849 20305637 PMC2851844

[21] B. E. Mierzwa *et al.*, “ Dynamic subunit turnover in ESCRT-III assemblies is regulated by Vps4 to mediate membrane remodelling during cytokinesis,” Nat. Cell Biol. 19, 787–798 (2017). 10.1038/ncb3559 28604678 PMC5493987

[22] B. Yang , G. Stjepanovic , Q. Shen , A. Martin , and J. H. Hurley , “ Vps4 disassembles an ESCRT-III filament by global unfolding and processive translocation,” Nat. Struct. Mol. Biol. 22, 492–498 (2015). 10.1038/nsmb.3015 25938660 PMC4456219

[23] G. van Niel *et al.*, “ The tetraspanin CD63 regulates ESCRT-independent and dependnent endosomal sorting during melanogenesis,” Dev. Cell 21, 708–721 (2011). 10.1016/j.devcel.2011.08.019 21962903 PMC3199340

[24] E. Gulbins and R. Kolesnick , “ Raft ceramide in molecular medicine,” Oncogene 22, 7070–7077 (2003). 10.1038/sj.onc.1207146 14557812

[25] N. Latycheva *et al.*, “ Syntenin-1 is a new component of tetraspanin-enriched microdomains: Mechanisms and consquences of the interaction of syntenin-1 with CD63,” Mol. Cell Biol. 26, 7707–7718 (2006). 10.1128/MCB.00849-06 16908530 PMC1636879

[26] B. Roucourt , S. Meeussen , J. Bao , P. Zimmermann , and G. David , “ Heparanase activates the syndecan-syntenin-ALIX exosome pathway,” Cell Res. 25, 412–428 (2015). 10.1038/cr.2015.29 25732677 PMC4387558

[27] E. Willms *et al.*, “ Cells release subpopulations of exosomes with distinct molecular and biological properties,” Sci. Rep. 6, 22519 (2016). 10.1038/srep22519 26931825 PMC4773763

[28] J. Escola *et al.*, “ Selective enrichment of tetraspan proteins on the internal vesicles of multivesicular endosomes and on exosomes secreted by human B-lymphocytes,” J. Biol. Chem. 273, 20121–20127 (1998). 10.1074/jbc.273.32.20121 9685355

[29] H. Boussion and N. Chaput , “ Les exosomes en practique clinique: Exemple du cancer bronchique,” Oncologie 17, 372–378 (2015). 10.1007/s10269-015-2545-9

[30] M. Li *et al.*, “ Analysis of the RNA content of exosomes derived from blood serum and urine and its potential as biomarkers,” Philos. Trans. R. Soc. B 369, 20130502 (2014). 10.1098/rstb.2013.0502 PMC4142023 25135963

[31] T. Janas , M. M. Janas , K. Sapon , and T. Janas , “ Mechanisms of RNA loading into exosomes,” FEBS Lett. 589, 1391–1398 (2015). 10.1016/j.febslet.2015.04.036 25937124

[32] C. Villarroya-Bltri *et al.*, “ Sumoylated hnRNPA2B1 controls the sorting of miRNA into exosomes through binding to specific motifs,” Nat. Commun. 4, 2980 (2013). 10.1038/ncomms3980 24356509 PMC3905700

[33] M. F. Bolukbasi *et al.*, “ miR-1289 and ‘Zipcode’-like sequence enrich mRNA in microvesicles,” Mol. Ther.–Nucl. Acids 1, e10 (2012). 10.1038/mtna.2011.2 PMC3381601 23344721

[34] E. R. Abels and X. O. Breakfield , “ Introduction to extracellular vesicles: Biogenesis, RNA cargo selection, content, and uptake,” Cell Mol. Neurobiol. 36, 301–312 (2016). 10.1007/s10571-016-0366-z 27053351 PMC5546313

[35] H. Valadi *et al.*, “ Exosome-mediated transfer of mRNAs and microRNAs is a novel mechanism of genetic exchange between cells,” Nat. Cell Biol. 9, 654–659 (2007). 10.1038/ncb1596 17486113

[36] J. Zhang *et al.*, “ Exosome and exosomal microRNA: Trafficking, sorting, and function,” Genomics Proeomics Bioinf. 13, 17–24 (2015). 10.1016/j.gpb.2015.02.001 PMC4411500 25724326

[37] V. Ramakrishnaiah *et al.*, “ Exosome-mediated transmission of hepatitis C virus between human hepatoma Huh7.5 cells,” Proc. Natl. Acad. Sci. U. S. A. 110, 13109–13113 (2013). 10.1073/pnas.1221899110 23878230 PMC3740869

[38] J. E. K. Hildreth , “ HIV as Trojan exosome: Immunological paradox explained?,” Front. Immunol. 8, 1715 (2017). 10.3389/fimmu.2017.01715 29250079 PMC5716971

[39] C. Subra , K. Laulagnier , B. Perret , and M. Record , “ Exosome lipidomics unravels lipid sorting at the level of multivesicular bodies,” Biochemie 89, 205–212 (2007). 10.1016/j.biochi.2006.10.014 17157973

[40] R. A. Haraszri *et al.*, “ High-resolution proteomic and lipidomic analysis of exosomes and microvesicles from different cell sources,” J. Extracell. Vesicles 5, 32570 (2016). 10.3402/jev.v5.32570 27863537 PMC5116062

[41] C. Villarroya-Bltri *et al.*, “ ISGylation controls exosomes secretion by promoting lysosomal degradation of MVB proteins,” Nat. Commun. 7, 13588 (2016). 10.1038/ncomms13588 27882925 PMC5123068

[42] L. Gangoda and S. Mathivanan , “ Cortactin enhances exosome secretion without altering cargo,” J. Cell Biol. 214, 129 (2016). 10.1083/jcb.201606131 27432895 PMC4949455

[43] N. P. Hessvik and A. Llorente , “ Current knowledge on exosome biogenesis and release,” Cell. Mol. Life Sci. 75, 193–208 (2018). 10.1007/s00018-017-2595-9 28733901 PMC5756260

[44] Y. Zhen and H. Stenmark , “ Cellular functions of Rab GTPases at a glance,” J. Cell Sci. 128, 3171–3176 (2015). 10.1242/jcs.166074 26272922

[45] L. Blanc and M. Vidal , “ New insights into the function of Rab GTPases in the context of exosomal secretion,” Small GTPases 9, 95–106 (2018). 10.1080/21541248.2016.1264352 28135905 PMC5902209

[46] C. Hsu *et al.*, “ Regulation of exosome secretion by Rab35 and its GTPase-activating proteins TBV1D10A-C,” J. Cell Biol. 189, 223–232 (2010). 10.1083/jcb.200911018 20404108 PMC2856897

[47] X. Yu , R. Prekeris , and G. Gould , “ Role of endosomal Rab GTPases in cytokinesis,” Eur. J. Cell Biol. 86, 25–35 (2017). 10.1016/j.ejcb.2006.10.002 17157409

[48] M. Ostrowski *et al.*, “ Rab27a and Rab27b control different steps of the exosome secretion pathway,” Nat. Cell Biol. 12, 19–30 (2010). 10.1038/ncb2000 19966785

[49] C. Chen *et al.*, “ Elucidation of exosome migration across the blood-brain barrier model in vitro,” Cell Mol. Bioeng. 9, 509–529 (2016). 10.1007/s12195-016-0458-3 28392840 PMC5382965

[50] Advisory Committee, Canadian Cancer Statistics, 2018.

[51] Y. H. Soung *et al.*, “ Exosomes in cancer diagnostics,” Cancer (Basal) 9, 8 (2017). 10.3390/cancers9010008 PMC5295779 28085080

[52] B. Sandfeld-Paulsen *et al.*, “ Exosomal proteins as prognostic biomarkers in non-small cell lung cancer,” Mol. Oncol. 10, 1595–1602 (2016). 10.1016/j.molonc.2016.10.003 27856179 PMC5423137

[53] Y. Chen *et al.*, “ Protein content and functional characteristics of serum-purified exosomes from patients with colorectal cancer revealed by quantitative proteomics,” Int. J. Cancer 140, 900–913 (2016). 27813080 10.1002/ijc.30496

[54] L. Rajendran *et al.*, “ Alzheimer's disease β-amyloid peptides are released in associated with exosomes,” PNAS 103, 11172–11177 (2006). 10.1073/pnas.0603838103 16837572 PMC1544060

[55] S. Ebrahimkhani *et al.*, “ Exosomal microRNA signatures in multiple sclerosis reflect disease status,” Sci. Rep. 7, 14293 (2017). 10.1038/s41598-017-14301-3 29084979 PMC5662562

[56] M. Garcia-Contreras *et al.*, “ Plasma-derived exosomes characterization reveals a dsitinct microRNA signature in long duration Type 1 diabetes,” Sci. Rep. 7, 5998 (2017). 10.1038/s41598-017-05787-y 28729721 PMC5519761

[57] M. Garcia-Contreras *et al.*, “ Exosomes as biomarkers and therapeutic tools for type1 diabetes mellitus,” Eur. Rev. Med. Pharmacol. Sci. 21, 2940–2956 (2017). 28682421

[58] W. Ying *et al.*, “ Adipose tissue macrophage-derived exosomal mirnas can modulate in vivo and in vitro insulin sensitivity,” Cell 171, 372–384.e12 (2017). 10.1016/j.cell.2017.08.035 28942920

[59] P. Li *et al.*, “ Progress in exosome isolation techniques,” Theranostics 7, 789–804 (2017). 10.7150/thno.18133 28255367 PMC5327650

[60] Y. Zhang and X.-F. Wang , “ A niche role for cancer exosomes in metastasis,” Nat. Cell Biol. 17, 709–711 (2015). 10.1038/ncb3181 26022917

[61] J. Maia , S. Caja , M. C. Strano Moraes , N. Couto , and B. Costa-Silva , “ Exosome-based cell-cell communication in the tumor microenvironment,” Front. Cell Dev. Biol. 6, 18 (2018). 10.3389/fcell.2018.00018 29515996 PMC5826063

[62] B. Costa-Silva *et al.*, “ Pancreatic cancer exosomes initiate pre-metastatic niche formation in the liver,” Nat. Cell Biol. 17, 816–826 (2015). 10.1038/ncb3169 25985394 PMC5769922

[63] V. Hyenne , O. Lefebvre , and J. G. Goetz , “ Going live with tumor exosomes and microvesicles,” Cell Adhes. Migr. 11, 173–186 (2017). 10.1080/19336918.2016.1276694 PMC5365253 28135898

[64] C. Roma-Rodrigues *et al.*, “ Tumor microenvironment modulation via gold nanoparticles targeting malicious exosomes: Implications for cancer diagnostics and therapy,” Int. J. Mol. Sci. 18, 162 (2017). 10.3390/ijms18010162 PMC5297795 28098821

[65] K. Meehan and L. J. Vella , “ The contribution of tumour-derived exosomes to the hallmarks of cancer,” Crit. Rev. Clin. Lab. Sci. 53, 121–131 (2016). 10.3109/10408363.2015.1092496 26479834

[66] J.-L. Qu *et al.*, “ Gastric cancer exosomes promote tumour cell proliferation through PI3K/Akt and MAPK/ERK activation,” Dig. Liver Dis. 41, 875–880 (2009). 10.1016/j.dld.2009.04.006 19473897

[67] C. Li , “ CD97 promotes gastric cancer cell proliferation and invasion through exosome-mediated MAPK signaling pathway,” World J. Gastroenterol. 21, 6215 (2015). 10.3748/wjg.v21.i20.6215 26034356 PMC4445098

[68] L. Yang , X.-H. Wu , D. Wang , C.-L. Luo , and L.-X. Chen , “ Bladder cancer cell-derived exosomes inhibit tumor cell apoptosis and induce cell proliferation in vitro,” Mol. Med. Rep. 8, 1272–1278 (2013). 10.3892/mmr.2013.1634 23969721

[69] S. Sento , E. Sasabe , and T. Yamamoto , “ Application of a persistent heparin treatment inhibits the malignant potential of oral squamous carcinoma cells induced by tumor cell-derived exosomes,” PLoS One 11, e0148454 (2016). 10.1371/journal.pone.0148454 26849680 PMC4743844

[70] J. Webber , R. Steadman , M. D. Mason , Z. Tabi , and A. Clayton , “ Cancer exosomes trigger fibroblast to myofibroblast differentiation,” Cancer Res. 70, 9621–9630 (2010). 10.1158/0008-5472.CAN-10-1722 21098712

[71] J. P. Webber *et al.*, “ Differentiation of tumour-promoting stromal myofibroblasts by cancer exosomes,” Oncogene 34, 290–302 (2015). 10.1038/onc.2013.560 24441045

[72] R. Kalluri , “ The biology and function of fibroblasts in cancer,” Nat. Rev. Cancer 16, 582–598 (2016). 10.1038/nrc.2016.73 27550820

[73] R. Kalluri and M. Zeisberg , “ Fibroblasts in cancer,” Nat. Rev. Cancer 6, 392–401 (2006). 10.1038/nrc1877 16572188

[74] R. C. Goulet *et al.*, “ Exosomes induce fibroblast differentiation into cancer-associated fibroblasts through TGFβ signaling,” Mol. Cancer Res. 16, 1196–1204 (2018). 10.1158/1541-7786.MCR-17-0784 29636362

[75] D. Todorova , S. Simoncini , R. Lacroix , F. Sabatier , and F. Dignat-George , “ Extracellular vesicles in angiogenesis,” Circ. Res. 120, 1658–1673 (2017). 10.1161/CIRCRESAHA.117.309681 28495996 PMC5426696

[76] M. F. Ribeiro , H. Zhu , R. W. Millard , and G.-C. Fan , “ Exosomes function in pro- and anti-angiogenesis,” Curr. Angiogenes 2, 54 (2014). 10.2174/22115528113020020001 PMC4217212 25374792

[77] T. Umezu , K. Ohyashiki , M. Kuroda , and J. H. Ohyashiki , “ Leukemia cell to endothelial cell communication via exosomal miRNAs,” Oncogene 32, 2747–2755 (2013). 10.1038/onc.2012.295 22797057

[78] S. Sharghi-Namini , E. Tan , L.-L. S. Ong , R. Ge , and H. H. Asada , “ Dll4-containing exosomes induce capillary sprout retraction in a 3D microenvironment,” Sci. Rep. 4, 4031 (2015). 10.1038/srep04031 PMC3916896 24504253

[79] F. van Zijl , G. Krupitza , and W. Mikulits , “ Initial steps of metastasis: Cell invasion and endothelial transmigration,” Mutat. Res. Mutat. Res. 728, 23–34 (2011). 10.1016/j.mrrev.2011.05.002 21605699 PMC4028085

[80] T. R. Geiger and D. S. Peeper , “ Metastasis mechanisms,” Biochim. Biophys. Acta BBA 1796, 293–308 (2009). 10.1016/j.bbcan.2009.07.006 19683560

[81] R. Singh , R. Pochampally , K. Watabe , Z. Lu , and Y.-Y. Mo , “ Exosome-mediated transfer of miR-10b promotes cell invasion in breast cancer,” Mol. Cancer 13, 256 (2014). 10.1186/1476-4598-13-256 25428807 PMC4258287

[82] K. Nakamura *et al.*, “ Exosomes promote ovarian cancer cell invasion through transfer of CD44 to peritoneal mesothelial cells,” Mol. Cancer Res. 15, 78–92 (2017). 10.1158/1541-7786.MCR-16-0191 27758876

[83] W. Zhou *et al.*, “ Cancer-secreted miR-105 destroys vascular endothelial barriers to promote metastasis,” Cancer Cell 25, 501–515 (2014). 10.1016/j.ccr.2014.03.007 24735924 PMC4016197

[84] H. Peinado *et al.*, “ Pre-metastatic niches: Organ-specific homes for metastases,” Nat. Rev. Cancer 17, 302–317 (2017). 10.1038/nrc.2017.6 28303905

[85] M. P. Plebanek *et al.*, “ Pre-metastatic cancer exosomes induce immune surveillance by patrolling monocytes at the metastatic niche,” Nat. Commun. 8, 1319 (2017). 10.1038/s41467-017-01433-3 29105655 PMC5673063

[86] J. L. Hood , R. S. San , and S. A. Wickline , “ Exosomes released by melanoma cells prepare sentinel lymph nodes for tumor metastasis,” Cancer Res. 71, 3792–3801 (2011). 10.1158/0008-5472.CAN-10-4455 21478294

[87] B. J. C. Quah and H. C. O'Neill , “ The immunogenicity of dendritic cell-derived exosomes,” Blood Cells, Mol., Dis. 35, 94–110 (2005). 10.1016/j.bcmd.2005.05.002 15975838

[88] G. Chen *et al.*, “ Exosomal PD-L1 contributes to immunosuppression and is associated with anti-PD-1 response,” Nature 560, 382 (2018). 10.1038/s41586-018-0392-8 30089911 PMC6095740

[89] C. Yang , S.-H. Kim , N. R. Bianco , and P. D. Robbins , “ Tumor-derived exosomes confer antigen-specific immunosuppression in a murine delayed-type hypersensitivity model,” PLoS One 6, e22517 (2011). 10.1371/journal.pone.0022517 21829629 PMC3149056

[90] C. Théry , M. Ostrowski , and E. Segura , “ Membrane vesicles as conveyors of immune responses,” Nat. Rev. Immunol. 9, 581–593 (2009). 10.1038/nri2567 19498381

[91] J. Wolfers *et al.*, “ Tumor-derived exosomes are a source of shared tumor rejection antigens for CTL cross-priming,” Nat. Med. 7, 297–303 (2001). 10.1038/85438 11231627

[92] F. M. Barros , F. Carneiro , J. C. Machado , and S. A. Melo , “ Exosomes and immune response in cancer: Friends or foes?,” Front. Immunol. 9, 730 (2018). 10.3389/fimmu.2018.00730 29696022 PMC5904196

[93] G. G. Romagnoli , B. B. Zelante , P. A. Toniolo , I. K. Migliori , and J. A. M. Barbuto , “ Dendritic cell-derived exosomes may be a tool for cancer immunotherapy by converting tumor cells into immunogenic targets,” Front. Immunol. 5, 692 (2015). 10.3389/fimmu.2014.00692 25646096 PMC4298225

[94] K. Essandoh *et al.*, “ Blockade of exosome generation with GW4869 dampens the sepsis-induced inflammation and cardiac dysfunction,” Biochim. Biophys. Acta 1852, 2362–2371 (2015). 10.1016/j.bbadis.2015.08.010 26300484 PMC4581992

[95] X. Qin *et al.*, “ Targeting Rabs as a novel therapeutic strategy for cancer therapy,” Drug Discovery Today 22, 1139–1147 (2017). 10.1016/j.drudis.2017.03.012 28390930

[96] L. Alvarez-Erviti *et al.*, “ Delivery of siRNA to the mouse brain by systemic injection of targeted exosomes,” Nat. Biotechnol. 29, 341–345 (2011). 10.1038/nbt.1807 21423189

[97] S. Ohno *et al.*, “ Systemically injected exosomes targeted to EGFR deliver antitumor microRNA to breast cancer cells,” Mol. Ther. 21, 185–191 (2013). 10.1038/mt.2012.180 23032975 PMC3538304

[98] Y. Tian *et al.*, “ A doxorubicin delivery platform using engineered natural membrane vesicle exosomes for targeted tumor therapy,” Biomaterials 35, 2383–2390 (2014). 10.1016/j.biomaterials.2013.11.083 24345736

[99] S. Taverna *et al.*, “ Curcumin modulates chronic myelogenous leukemia exosomes composition and affects angiogenic phenotype via exosomal miR-21,” Oncotarget 7, 30420–30439 (2016). 27050372 10.18632/oncotarget.8483 PMC5058690

[100] R. C. Piper and D. J. Katzmann , “ Biogenesis and function of multivesicular bodies,” Annu. Rev. Cell Dev. Biol. 23, 519–547 (2007). 10.1146/annurev.cellbio.23.090506.123319 17506697 PMC2911632

[101] H. Asai *et al.*, “ Depletion of microglia and inhibition of exosome synthesis halt tau propagation,” Nat. Neurosci. 18, 1584–1593 (2015). 10.1038/nn.4132 26436904 PMC4694577

[102] A. Savina , C. M. Fader , M. T. Damiani , and M. I. Colombo , “ Rab11 promotes docking and fusion of multivesicular bodies in a calcium-dependent manner: Ca2+-dependent multivesicular body fusion,” Traffic 6, 131–143 (2005). 10.1111/j.1600-0854.2004.00257.x 15634213

[103] Y.-D. Chen *et al.*, “ Exophagy of annexin A2 via RAB11, RAB8A and RAB27A in IFN-γ-stimulated lung epithelial cells,” Sci. Rep. 7, 5676 (2017). 10.1038/s41598-017-06076-4 28720835 PMC5516008

[104] W. Li *et al.*, “ Rab27A regulates exosome secretion from lung adenocarcinoma cells A549: Involvement of EPI64,” APMIS 122, 1080–1087 (2014). 24673604 10.1111/apm.12261

[105] A. Bobrie *et al.*, “ Rab27a supports exosome-dependent and -independent mechanisms that modify the tumor microenvironment and can promote tumor progression,” Cancer Res. 72, 4920–4930 (2012). 10.1158/0008-5472.CAN-12-0925 22865453

[106] L. A. Mulcahy , R. C. Pink , and D. R. F. Carter , “ Routes and mechanisms of extracellular vesicle uptake,” J. Extracell. Vesicles 3, 24641 (2014). 10.3402/jev.v3.24641 PMC4122821 25143819

[107] H. C. Christianson , K. J. Svensson , T. H. van Kuppevelt , J.-P. Li , and M. Belting , “ Cancer cell exosomes depend on cell-surface heparan sulfate proteoglycans for their internalization and functional activity,” Proc. Natl. Acad. Sci. 110, 17380–17385 (2013). 10.1073/pnas.1304266110 24101524 PMC3808637

[108] E. Macia *et al.*, “ Dynasore, a cell-permeable inhibitor of dynamin,” Dev. Cell 10, 839–850 (2006). 10.1016/j.devcel.2006.04.002 16740485

[109] T. Kawamoto *et al.*, “ Tumor-derived microvesicles induce proangiogenic phenotype in endothelial cells via endocytosis,” PLoS One 7, e34045 (2012). 10.1371/journal.pone.0034045 22479517 PMC3316594

[110] I. Hazan-Halevy *et al.*, “ Cell-specific uptake of mantle cell lymphoma-derived exosomes by malignant and non-malignant B-lymphocytes,” Cancer Lett. 364, 59–69 (2015). 10.1016/j.canlet.2015.04.026 25933830 PMC4490183

[111] Y. Zhang , H. F. Chan , and K. W. Leong , “ Advanced materials and processing for drug delivery: The past and the future,” Adv. Drug Delivery Rev. 65, 104–120 (2013). 10.1016/j.addr.2012.10.003 PMC3565095 23088863

[112] Y. H. Bae and K. Park , “ Targeted drug delivery to tumors: Myths, reality and possibility,” J. Controlled Release 153, 198–205 (2011). 10.1016/j.jconrel.2011.06.001 PMC3272876 21663778

[113] R. A. Bader, and P. A. David, *Engineering Polymer Systems for Improved Drug Delivery* ( Wiley, 2014).

[114] H. Maeda , H. Nakamura , and J. Fang , “ The EPR effect for macromolecular drug delivery to solid tumors: Improvement of tumor uptake, lowering of systemic toxicity, and distinct tumor imaging in vivo,” Adv. Drug Delivery Rev. 65, 71–79 (2013). 10.1016/j.addr.2012.10.002 23088862

[115] H. Kobayashi , R. Watanabe , and P. L. Choyke , “ Improving conventional enhanced permeability and retention (EPR) effects; What is the appropriate target?,” Theranostics 4, 81–89 (2014). 10.7150/thno.7193 PMC3881228 24396516

[116] M. Upreti , A. Jyoti , and P. Sethi , “ Tumor microenvironment and nanotherapeutics,” Transl. Cancer Res. 2, 309 (2013). 10.3978/j.issn.2218-676X.2013.08.11 24634853 PMC3951160

[117] H. Lv , S. Zhang , B. Wang , S. Cui , and J. Yan , “ Toxicity of cationic lipids and cationic polymers in gene delivery,” J. Controlled Release 114, 100–109 (2006). 10.1016/j.jconrel.2006.04.014 16831482

[118] F. Danhier , O. Feron , and V. Préat , “ To exploit the tumor microenvironment: Passive and active tumor targeting of nanocarriers for anti-cancer drug delivery,” J. Controlled Release 148, 135–146 (2010). 10.1016/j.jconrel.2010.08.027 20797419

[119] X. Li , M. McTaggart , and C. Malardier-Jugroot , “ Synthesis and characterization of a pH responsive folic acid functionalized polymeric drug delivery system,” Biophys. Chem. 214–215, 17–26 (2016). 10.1016/j.bpc.2016.04.002 27183249

[120] M. Cataldi , C. Vigliotti , T. Mosca , M. Cammarota , and D. Capone , “ Emerging role of the spleen in the pharmacokinetics of monoclonal antibodies, nanoparticles and exosomes,” Int. J. Mol. Sci. 18, 1249 (2017). 10.3390/ijms18061249 PMC5486072 28604595

[121] K. M. Tsoi *et al.*, “ Mechanism of hard-nanomaterial clearance by the liver,” Nat. Mater. 15, 1212–1221 (2016). 10.1038/nmat4718 27525571 PMC5132626

[122] A. Hunter , “ Molecular hurdles in polyfectin design and mechanistic background to polycation induced cytotoxicity,” Adv. Drug Delivery Rev. 58, 1523–1531 (2006). 10.1016/j.addr.2006.09.008 17079050

[123] M. S. Kim *et al.*, “ Development of exosome-encapsulated paclitaxel to overcome MDR in cancer cells,” Nanomed. Nanotechnol. Biol. Med. 12, 655–664 (2016). 10.1016/j.nano.2015.10.012 PMC4809755 26586551

[124] N. Kosaka , H. Izumi , K. Sekine , and T. Ochiya , “ microRNA as a new immune-regulatory agent in breast milk,” Silence 1, 7 (2010). 10.1186/1758-907X-1-7 20226005 PMC2847997

[125] X. Zhu *et al.*, “ Comprehensive toxicity and immunogenicity studies reveal minimal effects in mice following sustained dosing of extracellular vesicles derived from HEK293T cells,” J. Extracell. Vesicles 6, 1324730 (2017). 10.1080/20013078.2017.1324730 28717420 PMC5505007

[126] J. M. Pitt *et al.*, “ Dendritic cell–derived exosomes for cancer therapy,” J. Clin. Invest. 126, 1224–1232 (2016). 10.1172/JCI81137 27035813 PMC4811123

[127] T. Yang *et al.*, “ Exosome delivered anticancer drugs across the blood-brain barrier for brain cancer therapy in danio rerio,” Pharm. Res. 32, 2003–2014 (2015). 10.1007/s11095-014-1593-y 25609010 PMC4520542

[128] C. C. Conwell and L. Huang , “ Recent progress in non-viral gene delivery,” in *Non-Viral Gene Therapy*, edited by Taira K., Kataoka K., and Niidome T. ( Springer-Verlag, 2005), pp. 3–10.

[129] E. Cevher , A. Demir , and E. Sefik , “ Gene delivery systems: Recent progress in viral and Non-Viral therapy,” in *Recent Advances in Novel Drug Carrier Systems*, edited by Sezer A. D. ( InTech, 2012).

[130] Y. Zhou *et al.*, “ Exosome-mediated small RNA delivery for gene therapy: Therapeutic exosomal small RNA,” Wiley Interdiscip. Rev. RNA 7, 758–771 (2016). 10.1002/wrna.1363 27196002

[131] A. Mizrak *et al.*, “ Genetically engineered microvesicles carrying suicide mRNA/protein inhibit schwannoma tumor growth,” Mol. Ther. 21, 101–108 (2013). 10.1038/mt.2012.161 22910294 PMC3538300

[132] S. El-Andaloussi *et al.*, “ Exosome-mediated delivery of siRNA in vitro and in vivo,” Nat. Protoc. 7, 2112–2126 (2012). 10.1038/nprot.2012.131 23154783

[133] D. Sun *et al.*, “ A novel nanoparticle drug delivery system: The anti-inflammatory activity of curcumin is enhanced when encapsulated in exosomes,” Mol. Ther. 18, 1606–1614 (2010). 10.1038/mt.2010.105 20571541 PMC2956928

[134] X. Luan *et al.*, “ Engineering exosomes as refined biological nanoplatforms for drug delivery,” Acta Pharmacol. Sin. 38, 754 (2017). 10.1038/aps.2017.12 28392567 PMC5520184

[135] X. Zhuang *et al.*, “ Treatment of brain inflammatory diseases by delivering exosome encapsulated anti-inflammatory drugs from the nasal region to the brain,” Mol. Ther. 19, 1769–1779 (2011). 10.1038/mt.2011.164 21915101 PMC3188748

[136] C. Théry , S. Amigorena , G. Raposo , and A. Clayton , “ Isolation and characterization of exosomes from cell culture supernatants and biological fluids,” Curr. Protoc. Cell Biol. 30, 3.22.1–3.22.29 (2006). 10.1002/0471143030.cb0322s30 18228490

[137] C. Gardiner *et al.*, “ Techniques used for the isolation and characterization of extracellular vesicles: Results of a worldwide survey,” J. Extracell. Vesicles 5, 32945 (2016). 10.3402/jev.v5.32945 27802845 PMC5090131

[138] M. Wu *et al.*, “ Isolation of exosomes from whole blood by integrating acoustics and microfluidics,” Proc. Natl. Acad. Sci. 114, 10584–10589 (2017). 10.1073/pnas.1709210114 28923936 PMC5635903

[139] Y. Yuana , J. Levels , A. Grootemaat , A. Sturk , and R. Nieuwland , “ Co-isolation of extracellular vesicles and high-density lipoproteins using density gradient ultracentrifugation,” J. Extracell. Vesicles 3, 23262 (2014). 10.3402/jev.v3.23262 PMC4090368 25018865

[140] T. Baranyai *et al.*, “ Isolation of exosomes from blood plasma: qualitative and quantitative comparison of ultracentrifugation and size exclusion chromatography methods,” PLoS One 10, e0145686 (2015). 10.1371/journal.pone.0145686 26690353 PMC4686892

[141] E. Willms , C. Cabañas , I. Mäger , M. J. A. Wood , and P. Vader , “ Extracellular vesicle heterogeneity: Subpopulations, isolation techniques, and diverse functions in cancer progression,” Front. Immunol. 9, 738 (2018). 10.3389/fimmu.2018.00738 29760691 PMC5936763

[142] M. Tkach , J. Kowal , and C. Théry , “ Why the need and how to approach the functional diversity of extracellular vesicles,” Philos. Trans. R. Soc., B 373, 20160479 (2018). 10.1098/rstb.2016.0479 PMC5717434 29158309

[143] M. Giulietti *et al.*, “ Exploring small extracellular vesicles for precision medicine in prostate cancer,” Front. Oncol. 8, 221 (2018). 10.3389/fonc.2018.00221 29951374 PMC6008382

[144] J. Caradec *et al.*, “ Reproducibility and efficiency of serum-derived exosome extraction methods,” Clin. Biochem. 47, 1286–1292 (2014). 10.1016/j.clinbiochem.2014.06.011 24956264

[145] S. S. Kanwar , C. J. Dunlay , D. M. Simeone , and S. Nagrath , “ Microfluidic device (ExoChip) for on-chip isolation, quantification and characterization of circulating exosomes,” Lab Chip 14, 1891–1900 (2014). 10.1039/C4LC00136B 24722878 PMC4134440

[146] C. Chen *et al.*, “ Microfluidic isolation and transcriptome analysis of serum microvesicles,” Lab Chip 10, 505–511 (2010). 10.1039/B916199F 20126692 PMC3136803

[147] S. Nahavandi *et al.*, “ Microfluidic platforms for biomarker analysis,” Lab Chip 14, 1496–1514 (2014). 10.1039/C3LC51124C 24663505

[148] J. Rho *et al.*, “ Magnetic nanosensor for detection and profiling of erythrocyte-derived microvesicles,” ACS Nano 7, 11227–11233 (2013). 10.1021/nn405016y 24295203 PMC3898036

[149] B. J. Tauro *et al.*, “ Comparison of ultracentrifugation, density gradient separation, and immunoaffinity capture methods for isolating human colon cancer cell line LIM1863-derived exosomes,” Methods 56, 293–304 (2012). 10.1016/j.ymeth.2012.01.002 22285593

[150] N. Zarovni *et al.*, “ Integrated isolation and quantitative analysis of exosome shuttled proteins and nucleic acids using immunocapture approaches,” Methods 87, 46–58 (2015). 10.1016/j.ymeth.2015.05.028 26044649

[151] E. Pariset , V. Agache , and A. Millet , “ Extracellular vesicles: Isolation methods,” Adv. Biosyst. 1, 1700040 (2017). 10.1002/adbi.201700040 32646152

[152] J. C. Contreras-Naranjo , H.-J. Wu , and V. M. Ugaz , “ Microfluidics for exosome isolation and analysis: Enabling liquid biopsy for personalized medicine,” Lab Chip 17, 3558–3577 (2017). 10.1039/C7LC00592J 28832692 PMC5656537

[153] A. Bobrie , M. Colombo , S. Krumeich , G. Raposo , and C. Théry , “ Diverse subpopulations of vesicles secreted by different intracellular mechanisms are present in exosome preparations obtained by differential ultracentrifugation,” J. Extracell. Vesicles 1, 18397 (2012). 10.3402/jev.v1i0.18397 PMC3760636 24009879

[154] M. Li *et al.*, “ An optimized procedure for exosome isolation and analysis using serum samples: Application to cancer biomarker discovery,” Methods 87, 26–30 (2015). 10.1016/j.ymeth.2015.03.009 25814440

[155] D. W. Greening , R. Xu , H. Ji , B. J. Tauro , and R. J. Simpson , “ A protocol for exosome isolation and characterization: evaluation of ultracentrifugation, density-gradient separation, and immunoaffinity capture methods,” in *Proteomic Profiling Methods Protocols* ( Springer, New York, 2015), pp. 179–209. 10.1007/978-1-4939-2550-6_15 25820723

[156] H. G. Lamparski *et al.*, “ Production and characterization of clinical grade exosomes derived from dendritic cells,” J. Immunol. Methods 270, 211–226 (2002). 10.1016/S0022-1759(02)00330-7 12379326

[157] S. Gholizadeh *et al.*, “ Microfluidic approaches for isolation, detection, and characterization of extracellular vesicles: Current status and future directions,” Biosens. Bioelectron. 91, 588–605 (2017). 10.1016/j.bios.2016.12.062 28088752 PMC5323331

[158] K. W. Witwer *et al.*, “ Standardization of sample collection, isolation and analysis methods in extracellular vesicle research,” J. Extracell. Vesicles 2, 20360 (2013). 10.3402/jev.v2i0.20360 PMC3760646 24009894

[159] J. Van Deun *et al.*, “ The impact of disparate isolation methods for extracellular vesicles on downstream RNA profiling,” J. Extracell. Vesicles 3, 24858 (2014). 10.3402/jev.v3.24858 PMC4169610 25317274

[160] F. Yang , X. Liao , Y. Tian , and G. Li , “ Exosome separation using microfluidic systems: Size‐based, immunoaffinity‐based and dynamic methodologies,” Biotechnol. J. 12, 1600699 (2017). 10.1002/biot.201600699 28166394

[161] R. Linares , S. Tan , C. Gounou , N. Arraud , and A. R. Brisson , “ High-speed centrifugation induces aggregation of extracellular vesicles,” J. Extracell. Vesicles 4, 29509 (2015). 10.3402/jev.v4.29509 26700615 PMC4689953

[162] H. G. Barth , G. D. Saunders , and R. E. Majors , “ The state of the art and future trends of size-exclusion chromatography packings and columns,” LC GC N Am. 30, 544–563 (2012).

[163] J. F. Quintana *et al.*, “ Extracellular Onchocerca-derived small RNAs in host nodules and blood,” Parasites Vectors 8, 58 (2015). 10.1186/s13071-015-0656-1 25623184 PMC4316651

[164] D. Müller , S. Cattaneo , F. Meier , R. Welz , and A. J. deMello , “ Nanoparticle separation with a miniaturized asymmetrical flow field-flow fractionation cartridge,” Front. Chem. 3, 45 (2015). 10.3389/fchem.2015.00045 26258119 PMC4510429

[165] L.-L. Yu *et al.*, “ A comparison of traditional and novel methods for the separation of exosomes from human samples,” BioMed Res. Int. 2018, 3634563 10.1155/2018/3634563 30148165 PMC6083592

[166] A. Cheruvanky *et al.*, “ Rapid isolation of urinary exosomal biomarkers using a nanomembrane ultrafiltration concentrator,” Am. J. Physiol. 292, F1657–F1661 (2007). 10.1152/ajprenal.00434.2006 PMC2271070 17229675

[167] A. N. Böing *et al.*, “ Single-step isolation of extracellular vesicles by size-exclusion chromatography,” J. Extracell. Vesicles 3, 23430 (2014). 10.3402/jev.v3.23430 PMC4159761 25279113

[168] S. Fekete , A. Beck , J.-L. Veuthey , and D. Guillarme , “ Theory and practice of size exclusion chromatography for the analysis of protein aggregates,” J. Pharm. Biomed. Anal. 101, 161–173 (2014). 10.1016/j.jpba.2014.04.011 24816223

[169] P. Hong , S. Koza , and E. S. Bouvier , “ Size-exclusion chromatography for the analysis of protein biotherapeutics and their aggregates,” J. Liq. Chromatogr. Relat. Technol. 35, 2923–2950 (2012). 10.1080/10826076.2012.743724 23378719 PMC3556795

[170] B. J. Tauro *et al.*, “ Two distinct populations of exosomes are released from LIM1863 colon carcinoma cell-derived organoids,” Mol. Cell. Proteomics 12, 587 (2012). 10.1074/mcp.M112.021303 23230278 PMC3591653

[171] E. Zeringer , T. Barta , M. Li , and A. V. Vlassov , “ Strategies for isolation of exosomes,” Cold Spring Harb. Protoc. 2015, 319 (2015). 10.1101/pdb.top074476 25834266

[172] P. Cutler , “ Immunoaffinity chromatography,” in *Protein Purification Protocols* ( Springer, 2004), pp. 167–177.

[173] J. Mallorquí *et al.*, “ Purification of erythropoietin from human plasma samples using an immunoaffinity well plate,” J. Chromatogr. B 878, 2117–2122 (2010). 10.1016/j.jchromb.2010.06.025 20630810

[174] F. Momen-Heravi *et al.*, “ Current methods for the isolation of extracellular vesicles,” Biol. Chem. 394, 1253–1262 (2013). 10.1515/hsz-2013-0141 23770532 PMC7075462

[175] Y. Weng *et al.*, “ Effective isolation of exosomes with polyethylene glycol from cell culture supernatant for in-depth proteome profiling,” Analyst 141, 4640–4646 (2016). 10.1039/C6AN00892E 27229443

[176] X. Wang , “ Isolation of extracellular vesicles from breast milk,” in *Extracellular Vesicles* ( Springer, 2017), pp. 351–353. 10.1007/978-1-4939-7253-1_28 28828670

[177] G. M. Whitesides , “ The origins and the future of microfluidics,” Nature 442, 368 (2006). 10.1038/nature05058 16871203

[178] A. Dietzel , “ A brief introduction to microfluidics,” in *Microsystems for Pharmatechnology* ( Springer, 2016), pp. 1–21.

[179] J. M. Ng , I. Gitlin , A. D. Stroock , and G. M. Whitesides , “ Components for integrated poly (dimethylsiloxane) microfluidic systems,” Electrophoresis 23, 3461–3473 (2002). 10.1002/1522-2683(200210)23:20<3461::AID-ELPS3461>3.0.CO;2-8 12412113

[180] P. N. Nge , C. I. Rogers , and A. T. Woolley , “ Advances in microfluidic materials, functions, integration, and applications,” Chem. Rev. 113, 2550–2583 (2013). 10.1021/cr300337x 23410114 PMC3624029

[181] S.-C. Guo , S.-C. Tao , and H. Dawn , “ Microfluidics-based on-a-chip systems for isolating and analysing extracellular vesicles,” J. Extracell. Vesicles 7, 1508271 (2018). 10.1080/20013078.2018.1508271 30151077 PMC6104604

[182] T. Salafi , K. K. Zeming , and Y. Zhang , “ Advancements in microfluidics for nanoparticle separation,” Lab Chip 17, 11–33 (2017). 10.1039/C6LC01045H 27830852

[183] K. Lee , H. Shao , R. Weissleder , and H. Lee , “ Acoustic purification of extracellular microvesicles,” ACS Nano 9, 2321–2327 (2015). 10.1021/nn506538f 25672598 PMC4373978

[184] R. T. Davies *et al.*, “ Microfluidic filtration system to isolate extracellular vesicles from blood,” Lab Chip 12, 5202–5210 (2012). 10.1039/c2lc41006k 23111789

[185] H.-K. Woo *et al.*, “ Exodisc for rapid, size-selective, and efficient isolation and analysis of nanoscale extracellular vesicles from biological samples,” ACS Nano 11, 1360–1370 (2017). 10.1021/acsnano.6b06131 28068467

[186] L.-G. Liang *et al.*, “ An integrated double-filtration microfluidic device for isolation, enrichment and quantification of urinary extracellular vesicles for detection of bladder cancer,” Sci. Rep. 7, 46224 (2017). 10.1038/srep46224 28436447 PMC5402302

[187] Z. Wang *et al.*, “ Ciliated micropillars for the microfluidic-based isolation of nanoscale lipid vesicles,” Lab Chip 13, 2879–2882 (2013). 10.1039/c3lc41343h 23743667 PMC3740541

[188] J. S. Dudani *et al.*, “ Rapid inertial solution exchange for enrichment and flow cytometric detection of microvesicles,” Biomicrofluidics 9, 014112 (2015). 10.1063/1.4907807 25713694 PMC4320146

[189] C. Liu *et al.*, “ Field-free isolation of exosomes from extracellular vesicles by microfluidic viscoelastic flows,” ACS Nano 11, 6968–6976 (2017). 10.1021/acsnano.7b02277 28679045

[190] J. C. Yeo *et al.*, “ Label-free extraction of extracellular vesicles using centrifugal microfluidics,” Biomicrofluidics 12, 024103 (2018). 10.1063/1.5019983 30867854 PMC6404916

[191] M. He , J. Crow , M. Roth , Y. Zeng , and A. K. Godwin , “ Integrated immunoisolation and protein analysis of circulating exosomes using microfluidic technology,” Lab Chip 14, 3773–3780 (2014). 10.1039/C4LC00662C 25099143 PMC4161194

[192] W. Connacher *et al.*, “ Micro/nano acoustofluidics: Materials, phenomena, design, devices, and applications,” Lab Chip 18, 1952–1996 (2018). 10.1039/C8LC00112J 29922774

[193] Y. Ai , C. K. Sanders , and B. L. Marrone , “ Separation of Escherichia coli bacteria from peripheral blood mononuclear cells using standing surface acoustic waves,” Anal. Chem. 85, 9126–9134 (2013). 10.1021/ac4017715 23968497 PMC3789253

[194] H. A. Nieuwstadt , R. Seda , D. S. Li , J. B. Fowlkes , and J. L. Bull , “ Microfluidic particle sorting utilizing inertial lift force,” Biomed. Microdev. 13, 97–105 (2011). 10.1007/s10544-010-9474-6 PMC6839764 20865451

[195] M. Kesimer *et al.*, “ Characterization of exosomes-like vesicles released from human tracheobronchial ciliated epithelium: A possible role in innate defense,” FASEB J. 23, 1858–1868 (2009). 10.1096/fj.08-119131 19190083 PMC2698655

[196] Y. Wu , W. Deng , and D. J. I. Klinke , “ Exosomes: Improved methods to characterise their morphology, RNA content, and surface protein biomarkers,” Analyst 140, 6631–6642 (2015). 10.1039/C5AN00688K 26332016 PMC4986832

[197] X. Chen , B. Zheng , and H. Liu , “ Optical and digital microscopic imaging techniques and application in pathology,” Anal. Cell Pathol. 34, 5–18 (2012). 10.3233/ACP-2011-0006 PMC3310926 21483100

[198] V. Sokolova , *et.*, “ Characterisation of exosomes derived from human cells by nanoparticle tracking analysis and scanning electron microscopy,” Colloids Surf., B 87, 146–150 (2011). 10.1016/j.colsurfb.2011.05.013 21640565

[199] R. A. Dragovic *et al.*, “ Sizing and phenotyping of cellular vesicles using Nanoparticle Tracking Analysis,” Nanpmedicine 7, 780–788 (2011). 10.1016/j.nano.2011.04.003 PMC3280380 21601655

[200] D. R. Wilson and J. J. Green , “ Nanoparticle tracking analysis for determination of hydrodynamic diameter, concentration, and zeta-potential of polyplex nanoparticles,” J. Biomed. Nanotechnol. 1570, 31–46 (2017). 10.1007/978-1-4939-6840-4 28238128

[201] V. Filipe , A. Hawe , and W. Jiskoot , “ Critical evaluation of nanoparticle tracking analysis (NTA) by nanosight for the measurement of nanoparticles an protein aggregates,” Pharm. Res. 27, 796–810 (2010). 10.1007/s11095-010-0073-2 20204471 PMC2852530

[202] R. Szatanek *et al.*, “ The methods of choice for extracellular vesicles (EVs) characterization,” Int. J. Mol. Sci. 18, 1153 (2017). 10.3390/ijms18061153 PMC5485977 28555055

[203] W. Anderson *et al.*, “ A comparative study of submicron particle sizing platforms: Accuracy, precision and resolution analysis of polydispersed particle size distributions,” J. Colloid Interface Sci. 405, 322–330 (2013). 10.1016/j.jcis.2013.02.030 23759321

[204] S. Encarnación *et al.*, “ Comparative proteomics using 2-D gel electrophoresis and mass spectrometry as tools to dissect stimulons and regulons in bacteria with sequenced or partially sequenced genomes,” Biol. Proced. Online 7, 117–135 (2005). 10.1251/bpo110 16145578 PMC1190382

[205] S. Kreimer *et al.*, “ Mass-spectrometry-based molecular characterization of extracellular vesicles: Lipidomics and proteomics,” J. Proteome Res. 14, 2367–2384 (2015). 10.1021/pr501279t 25927954

[206] C. Yang *et al.*, “ Comprehensive proteomics analysis of exosomes derived from human seminal plasma,” Andrology 5, 1007–1015 (2017). 10.1111/andr.12412 28914500 PMC5639412

[207] G. Pocsfalvi *et al.*, “ Mass spectrometry of extracellular vesicles: Mass spectrometry of extracellular vesicles,” Mass Spectrom. Rev. 35, 3–21 (2016). 10.1002/mas.21457 25705034

[208] A. Poliakov , M. Spilman , T. Dokland , C. L. Amling , and J. A. Mobley , “ Structural heterogeneity and protein composition of exosome-like vesicles (prostasomes) in human semen,” Prostate 69, 159–167 (2009). 10.1002/pros.20860 18819103

[209] A. Görg , W. Weiss , and M. J. Dunn , “ Current two-dimensional electrophoresis technology for proteomics,” Proteomics 4, 3665–3685 (2004). 10.1002/pmic.200401031 15543535

[210] A. Llorente *et al.*, “ Molecular lipidomics of exosomes released by PC-3 prostate cancer cells,” Biochim. Biophys. Acta BBA 1831, 1302–1309 (2013). 10.1016/j.bbalip.2013.04.011 24046871

[211] Z. Wang *et al.*, “ Proteomic analysis of urine exosomes by multidimensional protein identification technology (MudPIT),” Proteomics 12, 329–338 (2012). 10.1002/pmic.201100477 22106071 PMC3517144

[212] K. L. Schey , J. M. Luther , and K. L. Rose , “ Proteomics characterization of exosome cargo,” Methods 87, 75–82 (2015). 10.1016/j.ymeth.2015.03.018 25837312 PMC4591097

[213] J. Kowal *et al.*, “ Proteomic comparison defines novel markers to characterize heterogeneous populations of extracellular vesicle subtypes,” PNAS 113, E968–E977 (2016). 10.1073/pnas.1521230113 26858453 PMC4776515

[214] A. Zhao *et al.*, “ Cell-free RNA contentin ruine as a possible molecular diagnostic tool for clear cell renal cell carcinoma,” Int. J. Cancer 136, 2610–2615 (2015). 10.1002/ijc.29313 25379634

[215] C. Glinge *et al.*, “ Stability of circulating clood-based microRNAs—Pre-analytic methodological considerations,” PLoS One 12, e0167969 (2017). 10.1371/journal.pone.0167969 28151938 PMC5289450

[216] D. Koppers-Lalic *et al.*, “ Nontemplated nucleotide additions distinguish the small RNA composition in cells from exosomes,” Cell Rep. 8, 1649–1658 (2014). 10.1016/j.celrep.2014.08.027 25242326

[217] L. Cheng , “ Characterization and deep sequencing analysis of exosomal and non-exosomal miRNA in human ruine,” Kidney Int. 86, 433–444 (2014). 10.1038/ki.2013.502 24352158

[218] M. Guescini , S. Genedani , V. Stocchi , and L. F. Agnati , “ Astrocytes and Glioblastoma cells release exosomes carrying mtDNA,” J. Neural Transm. 117, 1–4 (2010). 10.1007/s00702-009-0288-8 19680595

[219] J. Schageman *et al.*, “ The complete exosome workflow solution: From isolation to characterization of RNA cargo,” Biomed. Res. Int. 2013, 253957 (2013). 24205503 10.1155/2013/253957 PMC3800616

[220] D. Enderle *et al.*, “ Characterization of RNA from exosomes and other extracellular vesicles isolated by a novel spin column-based method,” PLoS One 10, e0136133 (2015). 10.1371/journal.pone.0136133 26317354 PMC4552735

[221] G. W. Tan , A. S. B. Khoo , and L. P. Tan , “ Evaluation of extractin kits and RT-qPCR systems adapted to high-throughput platform for circulating miRNAs,” Sci. Rep. 5, 9430 (2015). 10.1038/srep09430 25800946 PMC4371102

[222] Y. Mo , R. Wan , and Q. Zhang , “ Applications of reverse transcription-PCR and real-time PCR in nanotoxicity research,” Methods Mol. Biol. 926, 99–112 (2016). 10.1007/978-1-62703-002-1_7 PMC5087796 22975959

[223] Y. Engelborghs and A. J. W. G. Visser , *Fluorescence Spectroscopy and Microscopy* ( Humana Press, 2014), Vol. 1076.

[224] A. P. Joy *et al.*, “ Proteome profiling of extracellular vesicles captured with the affinity peptide Vn96: Comparison of Laemmli and TRIzol© protein-extraction methods,” J. Extracell. Vesicles 7, 1438727 (2018). 10.1080/20013078.2018.1438727 29511462 PMC5827780

[225] S. Mathivanan *et al.*, “ Proteomics analysis of A33 immunoaffinity-purified exosomes released from the human colon tumor cell line LIM1215 reveals a tissue-specific protein signature,” Mol. Cell Proteomics 9, 197 (2009). 10.1074/mcp.M900152-MCP200 19837982 PMC2830834

[226] O. P. B. Wiklander *et al.*, “ Extracellular vesicle in vivo biodistribution is determined by cell source, route of administration and targeting,” J. Extracell. Vesicles 4, 26316 (2015). 10.3402/jev.v4.26316 25899407 PMC4405624

[227] C. Chen *et al.*, “ Imaging and intracellular tracking of cancer-derived exosomes using single-molecule localization-based super-resolution microscope,” ACS Appl. Mater. Interfaces 8, 25825–25833 (2016). 10.1021/acsami.6b09442 27617891

[228] A. Suetsugu *et al.*, “ Imaging exosome transfer from breast cancer cells to stroma at metastatic sites in orthotopic nude-mouse models,” Adv. Drug Delivery Rev. 65, 383–390 (2013). 10.1016/j.addr.2012.08.007 22921594

[229] T. Tian *et al.*, “ Dynamics of exosome internalization and trafficking,” J. Cell. Physiol. 228, 1487–1495 (2013). 10.1002/jcp.24304 23254476

[230] G. Kibria *et al.*, “ A rapid, automated surface protein profiling of single circulating exosomes in human blood,” Sci. Rep. 6, 36502 (2016). 10.1038/srep36502 27819324 PMC5098148

[231] C. M. Wilson *et al.*, “ Sortilin mediates the release and transfer of exosomes in concert with two tyrosine kinase receptors,” J. Cell Sci. 127, 3983–3997 (2014). 10.1242/jcs.149336 25037567

[232] C. P. Lai *et al.*, “ Visualization and tracking of tumour extracellular vesicle delivery and RNA translation using multiplexed reporters,” Nat. Commun. 6, 7029 (2015). 10.1038/ncomms8029 25967391 PMC4435621

[233] G. Crivat and J. W. Taraska , “ Imaging proteins inside cells with fluorescent tags,” Trends Biotechnol. 30, 8–16 (2012). 10.1016/j.tibtech.2011.08.002 21924508 PMC3246539

[234] G. H. Patterson , “ Fluorescence microscopy below the diffraction limit,” Semin. Cell Dev. Biol. 20, 886–893 (2009). 10.1016/j.semcdb.2009.08.006 19698798 PMC2784032

[235] J. S. Aaron and J. A. Timlin , “ Advanced optical imaging of endocytosis,” in *Molecular Regulation of Endocytosis*, edited by Ceresa B. ( InTech, 2012).

[236] M. B. Stone , S. A. Shelby , and S. L. Veatch , “ Super-resolution microscopy: Shedding light on the cellular plasma membrane,” Chem. Rev. 117, 7457–7477 (2017). 10.1021/acs.chemrev.6b00716 28211677 PMC5471115

[237] J. Biteen and K. A. Willets , “ Introduction: Super-resolution and single-molecule imaging,” Chem. Rev. 117, 7241–7243 (2017). 10.1021/acs.chemrev.7b00242 28610423

[238] R. Heintzmann and T. Huser , “ Super-resolution structured illumination microscopy,” Chem. Rev. 117, 13890–13908 (2017). 10.1021/acs.chemrev.7b00218 29125755

[239] J. Tam and D. Merino , “ Stochastic optical reconstruction microscopy (STORM) in comparison with stimulated emission depletion (STED) and other imaging methods,” J. Neurochem. 135, 643–658 (2015). 10.1111/jnc.13257 26222552

[240] J. R. Allen , S. T. Ross , and M. W. Davidson , “ Single molecule localization microscopy for superresolution,” J. Opt. 15, 094001 (2013). 10.1088/2040-8978/15/9/094001

[241] B. O. Leung and K. C. Chou , “ Review of super-resolution fluorescence microscopy for biology,” Appl. Spectrosc. 65, 967–980 (2011). 10.1366/11-06398 21929850

